# Defying salinity, drought, and pH extremes: A multifunctional rhizobacterium, *Burkholderia gladioli* ST3M-39a, matches fertilizer efficacy in wheat via phosphate solubilization

**DOI:** 10.1371/journal.pone.0332355

**Published:** 2026-10-09

**Authors:** Sudip Silwal, Asmita Shrestha, Shreejan Pokharel, Bignya Chandra Khanal, Ramesh Acharya, Gyanu Raj Pandey

**Affiliations:** 1 Shubham Biotech Nepal Private Limited, Chitwan, Nepal; 2 Biotechnological Research and Development Center Private Limited, Chitwan, Nepal; 3 National Biotechnology Research Center, Nepal Agricultural Research Council, Lalitpur, Nepal; ICAR National Bureau of Agriculturally Important Microorganism, INDIA

## Abstract

Global phosphorus scarcity and the environmental impacts of chemical fertilizers necessitate sustainable microbial alternatives for agriculture. We characterized *Burkholderia gladioli* ST3M-39a, a maize rhizosphere isolate, as a multifunctional plant growth-promoting rhizobacterium with exceptional climate resilience. The strain achieved rapid phosphate solubilization (177.96 ± 5.26 µg/mL within 24 h; molybdenum-antimony assay), zinc solubilization, and ammonia production, EPS production, produced stress-alleviating enzymes (cellulase and protease), and biosurfactant production. Crucially, it maintained robust growth and phosphate-mobilizing capacity under extreme abiotic stresses: pH 4.5–8.5, 7.5% NaCl salinity, and drought-mimicking low water activity (a_w_ 0.950, 32% sorbitol). The strain was non-hemolytic and non-pathogenic to plants. In wheat trials, ST3M-39a inoculation significantly increased the growth parameters (p < 0.05 vs. those of the uninoculated controls), resulting in 85–92% of the biomass stimulation observed with diammonium phosphate (DAP) fertilizer. This multifunctional stress tolerance, coupled with its near-fertilizer efficacy, positioned ST3M-39a as a transformative bioinoculant for degraded soils. Field validation of its agricultural deployment and ecological impact is now pivotal.

## 1. Introduction

Establishing a sustainable agricultural system is a paramount global challenge driven by rising food demands. Meeting these demands through increased crop yields risks indiscriminate agrichemical use, causing environmental degradation [1]. In addition to resource depletion, biodiversity loss, and climate change, several abiotic stresses, including salinity, drought, cold, and metal toxicity, severely impair crop growth, development, and productivity worldwide [2]. An alternative strategy leverages beneficial plant microbiomes, known as plant growth-promoting bacteria (PGPB). These bacteria help plants acquire essential nutrients, promote growth, modulate phytohormones, and increase stress tolerance, offering an economically viable solution with significant agronomic utility [3,4].

Phosphorus is an indispensable plant macronutrient essential for nucleic acid synthesis, cell division, and critical cellular processes, including photosynthesis, carbohydrate metabolism, energy production, redox homeostasis, and signal transduction [5–7]. Its deficiency remains a primary growth-limiting factor in terrestrial ecosystems, as soil solution concentrations of orthophosphate (the plant-available form) rarely exceed 10 µM despite substantial total soil phosphorus reserves [8]. Crucially, soil phosphorus pools are dominated by recalcitrant organic compounds (30–65% of total P) and inorganic phosphorus (35–70%) that rapidly precipitate with metal cations (Fe³ ⁺ , Al³ ⁺ , Ca²^+^). This results in fertilizer-derived phosphorus accumulating primarily in plant-unavailable forms, sustaining phosphorus limitation in agricultural soils despite repeated fertilization and consequently reducing agricultural productivity [9–11].

The widespread application of phosphate fertilizers results in low recovery efficiency (10–30%), with residual phosphorus either becoming fixed in soils or contaminating aquatic systems through runoff. This accelerates eutrophication and expands agriculture’s carbon footprint [12,13]. The accumulation of soil phosphates is estimated to be sufficient to sustain global crop production for at least a century. Despite these reserves, chemical fertilizer production and use continue unabated worldwide [14]. Economic projections indicate that commercially viable phosphate reserves may be depleted within 100–200 years at current extraction rates [15]. Fertilizer prices surged 292.7% between 2000–2022 due to crop production inflation and rising global food costs. Furthermore, mining and processing phosphate fertilizers, which contain natural radioactive elements, compromise public health [14]. Given the environmental damage from fertilizer overuse, underutilization of legacy phosphorus, and dwindling global reserves, developing sustainable strategies to enhance soil phosphorus bioavailability is imperative for food security [5].

Phosphate-solubilizing bacteria (PSB) offer a sustainable strategy to mitigate soil phosphorus deficiency by enhancing bioavailable phosphorus through three key mechanisms: (1) acid-mediated mineralization of fixed phosphorus pools, (2) enzymatic hydrolysis of organic compounds, and (3) chelation of metal ions (Fe³ ⁺ , Al³ ⁺ , Ca²^+^) via extracellular polysaccharides and organic acids. These processes collectively reduce the soil pH while liberating orthophosphates [16]. As biofertilizers, PSB enhance plant growth, nutritional status, root architecture, stress tolerance, and competitive ability [17]. Documented growth improvements in maize, wheat, paddy, rice, pea, and lettuce confirm their efficacy across diverse crops [18–21], with specific studies demonstrating significant increases in wheat biomass and phosphorus content following PSB inoculation [17]. In addition to phosphorus solubilization, these microorganisms function as phytohormone producers, biocontrol agents against pathogens, heavy-metal bioremediation agents, and abiotic stress mitigators [22].

Bacterial genera including *Pseudomonas*, *Bacillus*, *Rhizobium*, *Burkholderia*, *Azotobacter*, *Microbacterium*, and *Azospirillum* represent the most significant PSB [23,24]. In low-phosphorus soil, *Burkholderia* species enhance maize biomass by 15−30% and increase bioavailable phosphorus in the rhizosphere by 20−50% [25]. As high-performance PSB, *Burkholderia* species exhibit broad biotechnological potential, promoting plant growth and development while conferring resistance against biotic and abiotic stresses [26]. Notably, *B. gladioli* have robust plant growth-promoting effects through multiple mechanisms: phosphate solubilization, nitrogen fixation, indole-3-acetic acid (IAA) production, ACC (1-aminocyclopropane-1-carboxylate) deaminase activity, siderophore secretion, and phytohormone synthesis. These traits stem from its capacity to biosynthesize diverse bioactive secondary metabolites, positioning *B. gladioli* as a prime candidate for ecological fertilizer development [27–31].

This study isolated and characterized *Burkholderia gladioli* ST3M-39a from maize rhizosphere soil as a multifunctional plant growth-promoting rhizobacterium (PGPR) with high phosphate-solubilization activity. We systematically evaluated its: (i) phosphate solubilization efficiency under extreme abiotic stresses, (ii) other plant growth promoting traits, and (iii) functional impact on wheat growth in greenhouse trials, demonstrating near-fertilizer efficacy.

## 2. Materials and methods

### 2.1 Protocol availability

The protocols used in this study, titled ‘Defying salinity, drought, and pH extremes: a multifunctional rhizobacterium, *Burkholderia gladioli* ST3M-39a, matches fertilizer efficacy in wheat via phosphate solubilization’, are available in protocols.io with DOI: dx.doi.org/10.17504/protocols.io.yxmvm17j6v3p/v1.

### 2.2 Chemical and equipment

All microbiological growth media and general reagents were procured from HiMedia and Fisher Scientific. Molecular biology-grade reagents, buffers, and PCR master mix were sourced from Amresco and New England Biolabs, respectively, with PCR primers synthesized by Macrogen (South Korea). Laboratory glassware was supplied by Borosil. Microbial characterization employed an Olympus CX21i microscope. DNA extraction and other centrifugation utilized a REMI NEYA 16R centrifuge, PCR amplification was performed on a MiniAmp^TM^ Plus Thermal Cycler (Thermo Fisher Scientific), and spectrophotometric measurements used a Labdex LX111VS instrument.

### 2.3 Isolation and maintenance of organisms

Organisms were isolated from rhizospheric soil of maize plants collected in Dumre beshi, Bharatpur-29, Chitwan, Nepal (27°48’52”N and 084°28’47”E) as shown in S1 Fig. Soil samples were collected from privately owned farmland in the Chitwan district, Nepal, with verbal permission from the landowner. No specific permits were required for the collection of soil samples for microbiological research at this site, as the study did not involve endangered or protected species. The soil samples (1 g) were homogenized in 10 ml of sterile water, serially diluted, and spread onto yeast mannitol agar. The plates were incubated aerobically at 30°C for 24 h. Morphologically distinct bacterial colonies were subcultured on glucose-yeast extract-peptone (GYP; 1% glucose, 0.5% yeast extract, 0.3% peptone) agar to obtain pure cultures. Isolates were preserved as glycerol stocks at −80°C for long-term storage and maintained on bacterial slants (stored at 4°C) for experimental use [32].

### 2.4 Screening for phosphate solubilization

#### 2.4.1 Qualitative assay.

Isolates were screened for phosphate solubilization on Pikovskaya’s agar (PKA) [33] and National Botanical Research Institute’s phosphate (NBRIP) media [34]. Both media were incubated at 30°C for 72 h under aerobic conditions. Colonies that formed halo zones on both media were identified as phosphate solubilizers. Isolate ST3M-39a, the sole isolate exhibiting phosphate solubilization on both media, was selected for further analysis. The phosphate solubilization index (PSI) was calculated as follows:


$$\begin{array}{c} \text{PSI }=\frac{\text{Colony diameter }+\text{ halo region diameter}}{\text{colony diameter}} \end{array}$$

#### 2.4.2 Quantitative assay.

The strain exhibiting the sole halo zone was selected for quantitative phosphate solubilization analysis. The strain was precultured in nutrient broth (24 h, 30°C, 100 rpm) before the standardized inoculum (OD_600_ 0.6; 2–10% v/v) was aseptically transferred to 30 mL NBRIP medium (pH 6.8) in 250 mL flasks, with uninoculated medium used as a negative control. Cultures were incubated aerobically (30°C, 100 rpm), and 1 mL samples were collected after 24 h for phosphate quantification. After centrifugation (10,000 × g, 5 min, 4°C), the supernatant was analyzed for solubilized phosphate via the molybdenum-antimony colorimetric method [35]. The final pH of the medium was also recorded. All the experiments were performed in triplicate.

### 2.5 Identification of organism

#### 2.5.1 Molecular identification.

**2.5.1.1 DNA extraction and sequencing:** The bacterial isolate was incubated in nutrient broth at 30°C for 24 h. Genomic DNA was extracted via the Sambrook and Russell method [36], and the 16S rRNA gene was amplified with the universal primers 27F (5’-AGAGTTTGATCMTGGCTCAG-3’) and 1492R (5-TACGGYTACCTTGTTACGACTT-3’). Sequencing was performed by Macrogen (South Korea). The raw sequences were assembled and trimmed in a codon code aligner, and the consensus sequence was subsequently deposited in NCBI GenBank (accession OR143748.1). For phylogenetic analysis, the sequence was compared to the NCBI database via BLASTN (16S rRNA dataset), and 20 closely related sequences were retrieved in FASTA format.

**2.5.1.2 Maximum likelihood analysis of taxa:** A maximum likelihood phylogenetic tree was constructed in MEGA12 [37] via the Tamura-Nei model [38], with nodal support assessed via 2,000 bootstrap replicates [39].

#### 2.5.2 Morphological and biochemical characterization.

The isolate was identified morphologically and biochemically following *Bergey’s Manual of Systematic Bacteriology* [40].

### 2.6 Growth under different abiotic conditions

Bacterial growth was assessed in nutrient broth under controlled abiotic stresses: pH gradients (4.0–11.5; 0.5–1.0 increments), NaCl concentrations (0–9.0% w/v; pH 6.8), and sorbitol-induced osmotic stress (0–40% w/v; pH 6.8). Water activity (a_w_) for sorbitol solutions was calculated following established methods [41]. Overnight cultures (OD_600_ = 0.6) were aseptically inoculated at 2% (v/v) into fresh NBRIP medium supplemented with test conditions. Cultures underwent aerobic incubation at 30°C with 100 rpm agitation for 48 h. All treatments were replicated in triplicate.

### 2.7 Phosphate solubilization under different abiotic conditions

Phosphate solubilization was assessed in NBRIP medium under variable stress conditions: pHs (4.5, 7, and 8.5), NaCl (0–7.5% w/v, 2.5% increments; initial pH 6.8), and sorbitol-induced osmotic stress (0–32% w/v, 8% increments; initial pH 6.8). Overnight nutrient broth culture (OD_600_ = 0.6) was inoculated at 6% (v/v) into test media. Following 24 h aerobic incubation at 30°C with 100 rpm agitation, solubilized phosphate was quantified using previously described method and final pH recorded. All assays were performed in triplicate.

### 2.8 Other PGPR properties

#### 2.8.1 Zinc solubilization.

Zinc solubilization was assessed by culturing the isolate in mineral salt media supplemented with 0.1% zinc oxide [20]. The plates were incubated at 30°C for 7 days, and the zinc solubilization efficiency (ZSE%) was calculated as (clearing zone diameter/colony diameter) × 100 [42].

#### 2.8.2 Exopolysaccharide production.

Exopolysaccharide (EPS) production was induced by inoculating 100 mL LB broth with 100 μL bacterial culture (OD_600_ = 0.6) followed by 96 h incubation at 30°C with 100 rpm orbital shaking. Cells were pelleted by centrifugation, and the supernatant treated with two volumes of ice-cold 95% ethanol to precipitate EPS. Precipitates were recovered, dried, and quantified gravimetrically [43,44].

#### 2.8.3 Cellulase activity.

For cellulolytic activity, the isolates were grown in basal salt media supplemented with 1% carboxymethyl cellulose (30°C, 48 h). After incubation, the agar plate was stained with 0.1% Congo red solution for 15 min, followed by counterstaining with 1 M NaCl (20 min). Cellulose hydrolysis was identified by clear zones surrounding colonies [45].

#### 2.8.4 Protease activity.

For the protease test, the isolate was cultured on skim milk powder agar (5% skim milk powder) and incubated at 30°C for 48 hours. The clear halo region indicates the production of protease by the isolate [46].

#### 2.8.5 Ammonia production.

Ammonia production was qualitatively evaluated via the method by Chen *et al*. [47] by inoculating test strains in peptone broth and incubating them at 30°C for 48 h. Following incubation, 500 μL of Nessler’s reagent was added, with the development of a yellow to brown color indicating presence of ammonia.

#### 2.8.6 Biosurfactant detection assay.

The bacterial isolate was cultivated in a modified minimal salts medium (30°C, 180 rpm, 96 h) composed of (in g L^−1^): K_2_HPO_4_, 2.0; KH_2_PO_4_, 2.0; MgSO_4_·7H_2_O, 0.2; (NH_4_)_2_SO_4_, 1.0; glucose, 10.0; with trace elements CaCl_2_ and FeSO_4_·7H_2_O each at 0.001 g L^−1^, pH 7.0 [48]. Post-incubation, biomass was removed by centrifugation (10,000 × g, 15 min) and the cell-free supernatant was aseptically filtered (0.22 µm). Biosurfactant presence was assessed via a qualitative drop collapse assay, adapted from Bodour and Miller-Maier [49,50], wherein 2 µL of paraffin oil was equilibrated overnight at ambient temperature in a 96-well plate lid, followed by the application of 5 µL of clarified supernatant onto the oil surface; drop spreading or collapse was subsequently documented.

### 2.9 Safety assessment

#### 2.9.1 Plant pathogenicity assay.

A leaf spray assay was performed to assess the potential for plant disease caused by ST3M-39a. Bacterial cells were harvested and resuspended to an OD_600_ of 1.0. This suspension was sprayed onto the leaves of three-week-old bok choy (*Brassica rapa* subsp. *Chinensis*) plants, using a modified version of the method described by Wang *et al* [51]. Control plants were sprayed with nutrient broth. The experiment utilized three leaves from different plants per treatment as biological replicates. After 2 weeks, leaf tissues and mesophyll cells were examined for symptoms of damage using light microscopy. Leaf length, breadth, and root length were recorded. The experiment was conducted in four independent biological repeats.

#### 2.9.2 Hemolysis assay.

The hemolytic activity of ST3M-39a was tested by streaking the strain onto blood agar plates supplemented with 5% (v/v) defibrinated sheep blood [52]. Plates were incubated at 37°C for 24 hours and examined for zone of hemolysis.

### 2.10 Greenhouse experiment

A controlled pot trial was conducted using 45 sterile 9-inch diameter pots filled with autoclaved silty loam soil collected from the upper horizon (0–20 cm) of an agricultural research field at “Nepal Agricultural Research Council, Khumaltar, Nepal”. Representative soil from the same source site was previously characterized with the following average properties: pH 4.76, 2.65% organic matter, 163.35 mg/kg available phosphorus (extracted as P_2_O_5_), 012% total nitrogen, and 55.31 mg/kg available potassium (K_2_O). These values are provided as reference characteristics of the soil type used; a full analysis of the specific experimental batch was not performed. The wheat (*Triticum aestivum* ‘WK 1204’) was sown, and germination occurred one-week post-sowing. The seedlings were thinned to five uniform plantlets per pot and maintained under greenhouse conditions. The experimental design consisted of three treatments, each replicated 15 times (biological replicates): (A) a negative control (no amendments), (B) a positive control (diammonium phosphate [DAP] applied at a rate equivalent to 90 kg/ha), and (C) a bacterial treatment (1 mL of a *B. gladioli* ST3M-39a inoculum at 1 × 10^9^ CFU/mL). Throughout the growing season, each pot received 50 mL of autoclaved water weekly. This maize rhizosphere isolate was tested on wheat to assess its broader functional potential as a cross-crop plant growth-promoting rhizobacterium.

At physiological maturity, the following agronomic parameters were measured: shoot length, root length, spike length, chlorophyll content (using a SPAD-502 meter), dry biomass per pot (without spikes), grain yield per pot, and seed number per pot were measured [17].

### 2.11 Statistical analysis

All quantitative data were analyzed with Origin Pro software and organized using Microsoft Excel. Significance differences among treatment means were determined by one-way analysis of variance (ANOVA). Prior to ANOVA, the assumption of homogeneity of variance was assessed using Levene’s test. For datasets meeting this assumption (p > 0.05), standard one-way ANOVA was applied. When the assumption was violated (p < 0.05), the robust Welch’s ANOVA was used. For datasets where the ANOVA or Welch’s F-statistics were significant (p < 0.05), post hoc tests were used to identify specific differences between group means. Tukey’s HSD test was applied following a standard ANOVA, while the Games-Howell procedure was used following a significant Welch’s ANOVA, thereby maintaining a family-wise error rate of 5% in pairwise comparisons. For direct pairwise comparisons between treatment and control groups in the pathogenicity assay (leaf length, breadth, and root length), a two-tailed paired sample t-test was employed, testing the null hypothesis of no significant difference. All tests maintained a significance level of p < 0.05.

## 3. Results

### 3.1 Screening for phosphate solubilization

The tested isolate exhibited significant phosphate-solubilizing activity, as evidenced by halo zone formation on both Pikovskaya’s (PVK) and National Botanical Research Institute’s Phosphate (NBRIP) agar media (Fig 1A). The phosphate solubilization index (PSI) was comparable in both media, with values of 1.27 ± 0.097 (PVK) and 1.41 ± 0.10 (NBRIP), indicating efficient mineral phosphate solubilization.

**Fig 1:**
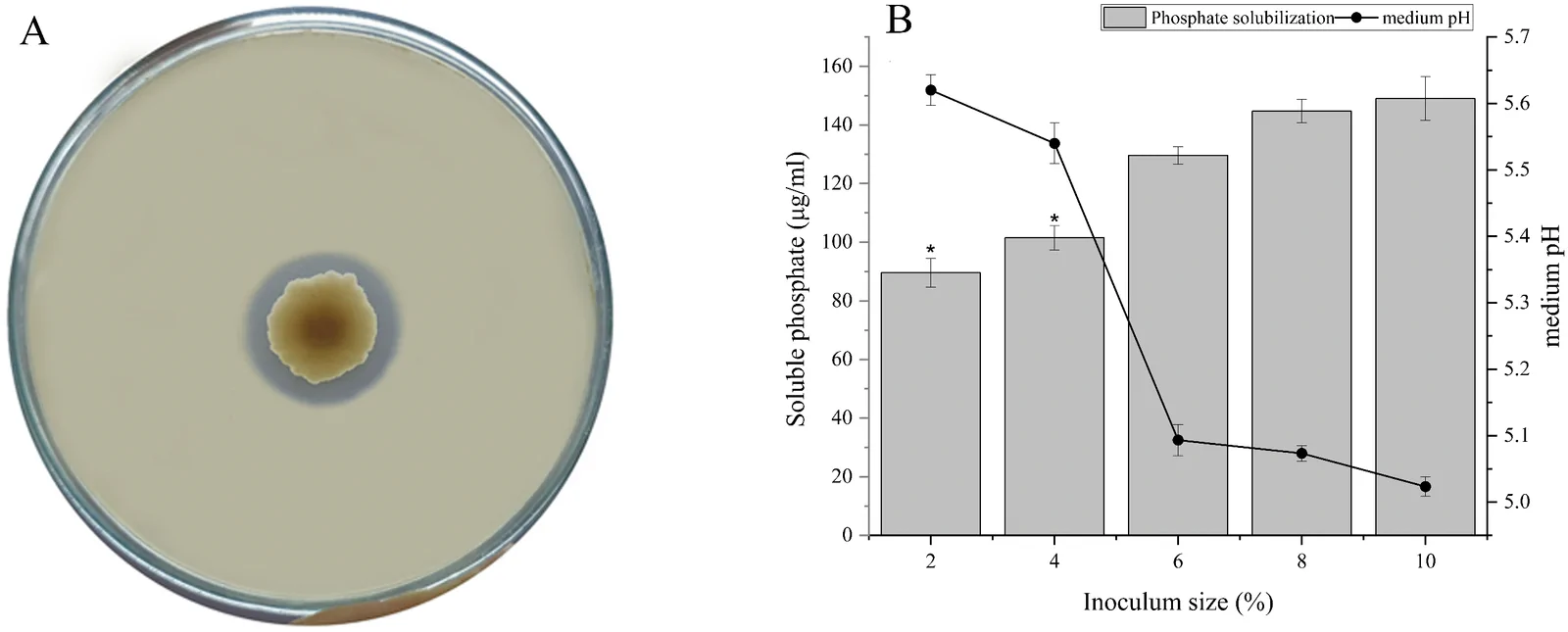
Assessment of phosphate solubilization by isolate ST3M-39a. (A) Formation of distinct halo zones around the colony on PVK agar. (B) Relationship between inoculum dose, soluble phosphate released, and final pH of NBRIP medium. *Significantly lower than 10% inoculum (α = 0.05). Error bars represent standard error (n = 3).

Further quantitative analysis revealed that inoculum size significantly influenced phosphate solubilization efficiency (Fig 1B). A one-way ANOVA was performed, with the assumption of homogeneity of variance confirmed by Levene’s test. The ANOVA revealed a significant main effect of inoculum size. The highest soluble phosphate concentration (148.97 ± 7.45 µg/mL) was achieved with a 10% inoculum. Post hoc comparisons showed this value was significantly greater (p < 0.05) than that obtained with the 4% (101.52 ± 4.13 µg/mL) and 2% (89.58 ± 4.93 µg/mL) inocula. However, since the phosphate solubilization ability of the 6% (129.65 ± 2.97 µg/mL) and 8% (144.71 ± 3.98 µg/mL) inocula was not significantly different from that of the 10% inoculum, the 6% inoculum was selected for subsequent experiments due to its resource efficiency.

The initial pH of the medium (pH 6.8) decreased after incubation. The most pronounced reduction (pH 5.02 ± 0.01) occurred with the 10% inoculum. The pH decline became less pronounced with decreasing inoculum size: 8% (pH 5.07 ± 0.01), 6% (pH 5.09 ± 0.02), 4% (pH 5.54 ± 0.03), and 2% (pH 5.62 ± 0.02), as shown in Fig 1B. However, the pH decline was similar for the 6%, 8%, and 10% inoculum sizes.

### 3.2 Identification of the organism

#### 3.2.1 Molecular characterization.

Comparisons using nucleotide BLAST of the 16S rRNA sequence of strain ST3M-39a revealed 99.81% identity with *Burkholderia gladioli* strain NBRC 13700, 99.53% identity with *Burkholderia plantarii* strain CIP 105769, 99.41% identity with *Burkholderia perseverans* strain INN12, and 99.16% identity with *Burkholderia glumae* strain P 1-22-1. Maximum likelihood analysis revealed that strain ST3M-39a clustered robustly within the *B. gladioli* monophyletic clade, exhibiting the closest phylogenetic affinity to reference strains of *B. gladioli*, with all strains forming a highly supported subclade (bootstrap > 95%) distinct from other *Burkholderia* species, including the phytopathogens *B. glumae* (LMG 2196) and *B. plantarii* (NIAES 1723), opportunistic pathogen *B. cenocepacia* (LMG 16656), and environmental species *B. stagnalis* (LMG 28156), as shown in Fig 2.

**Fig 2:**
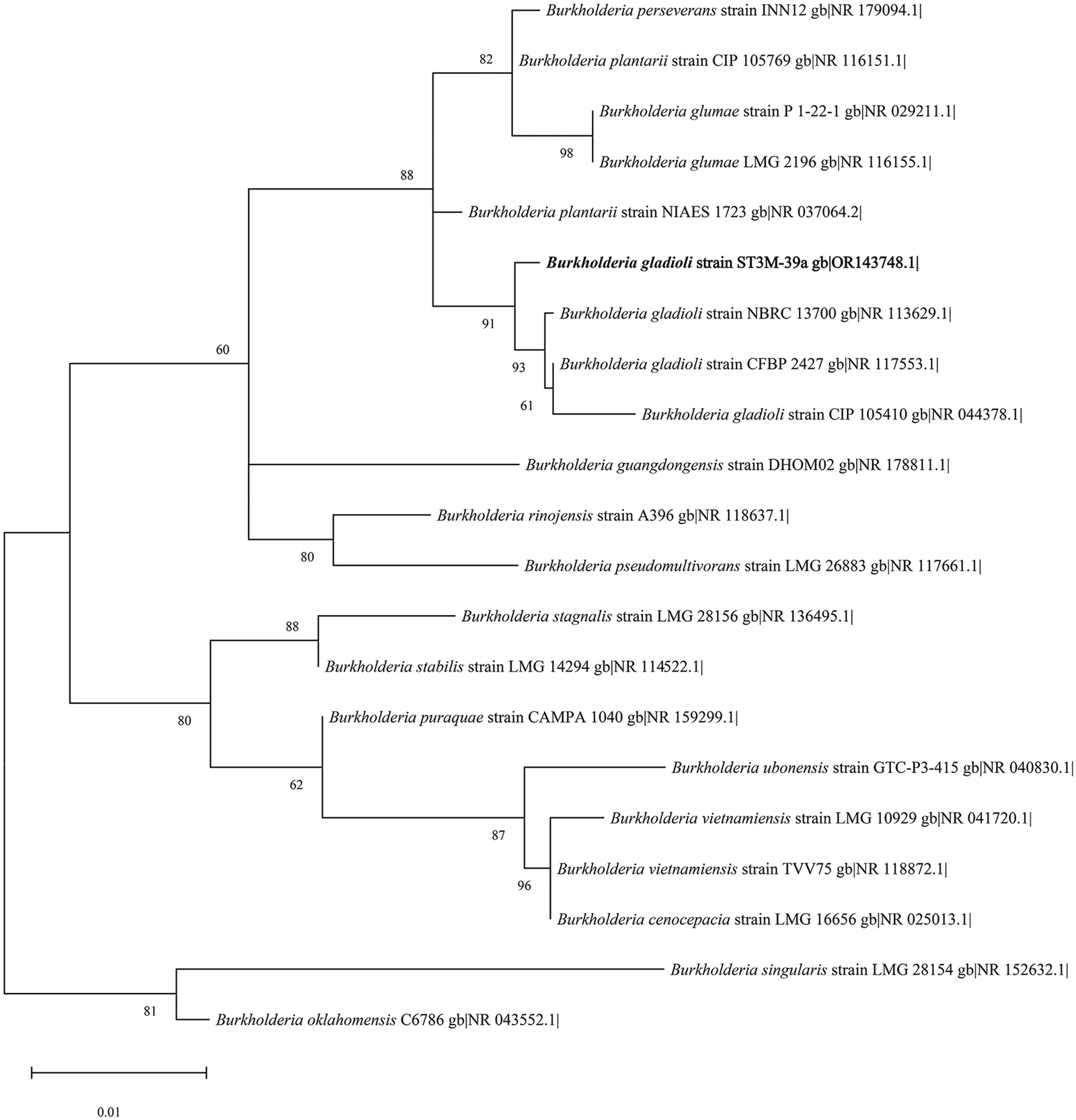
Maximum likelihood phylogenetic tree of the bacterial isolate based on 16S rRNA gene sequences. The tree was constructed using the Tamura-Nei model. Bootstrap values (2000 replicates) are shown at nodes; branches with less than 50% bootstrap support were collapsed. Initial topologies were generated using neighbor-joining and BioNJ algorithms, and the optimal tree was selected based on the highest log-likelihood score.

#### 3.2.2 Morphological and biochemical characterization.

The isolate ST3M-39a showed the typical morphological characteristics of Gram-negative rods. It produced colonies that were circular, raised, with entire margins, smooth and glistening surface, opaque in appearance and creamy white in pigmentation with a diameter of about 1.5 mm. Isolate was negative for Voges-Proskauer, indole production, motility, hydrogen sulfide production and oxidase but positive for catalase and oxidative metabolism in OF medium. It was positive for C4 lipase, gelatinase, protease, cellulase and urease and negative for amylase. The isolate grew well on LB media and MacConkey agar (pink colonies without precipitate), tolerated 37°C and 40°C (but not 45°C), pH 8 and 7% NaCl. It could use different carbon sources like glucose, sucrose, fructose, xylose, galactose, lactose, melezitose, sorbitol, ethanol, propanol, n-hexadecane, sodium-K-tartarate and citrate but not maltose, mannitol or pyruvate. It used the amino acids L-arginine, glycine, L-histidine, L-lysine, L-phenylalanine, L-threonine and L-tryptophan and L-glutamine, but not L-cysteine or L-ornithine. The isolate was resistant to ampicillin, penicillin, cefotaxime, vancomycin, rifampicin, cefuroxime and carbenicillin and sensitive to ciprofloxacin, gentamicin, moxifloxacin, levofloxacin, azithromycin and erythromycin (as shown in S1 Table).

### 3.3 Growth under different abiotic conditions

#### 3.3.1 pH levels.

The isolate ST3M-39a demonstrated measurable growth across a pH spectrum of 5–11 (Fig 3A). The assumption of homogeneity of variances was violated, as indicated by Levene’s test. Therefore, Welch’s one-way ANOVA was conducted, confirming a significant effect of pH on growth. Post hoc comparisons using the Games-Howell test revealed that growth at pH 7 (2.023 ± 0.059) was statistically equivalent to growth at pH 5.5 (2.108 ± 0.043), 6 (2.203 ± 0.077), 8 (1.998 ± 0.024), 9 (2.092 ± 0.044), and 10 (1.782 ± 0.047) (all p > 0.05), defining an optimal growth range of pH 5.5–10. Growth was significantly lower, though still detectable, at pH 4 (0.005 ± 0.002), 4.5 (0.048 ± 0.022), 5 (0.28 ± 0.008), 11 (0.307 ± 0.041), and 11.5 (0.052 ± 0.003) (all p < 0.05 versus pH 7). Notably, while growth was suboptimal, measurable biological activity was still present at pH 5 and 11. Therefore, while the optimal pH range for growth was defined as pH 5.5–10, the isolate maintained activity across a broader pH spectrum from 5 to 11.

**Fig 3:**
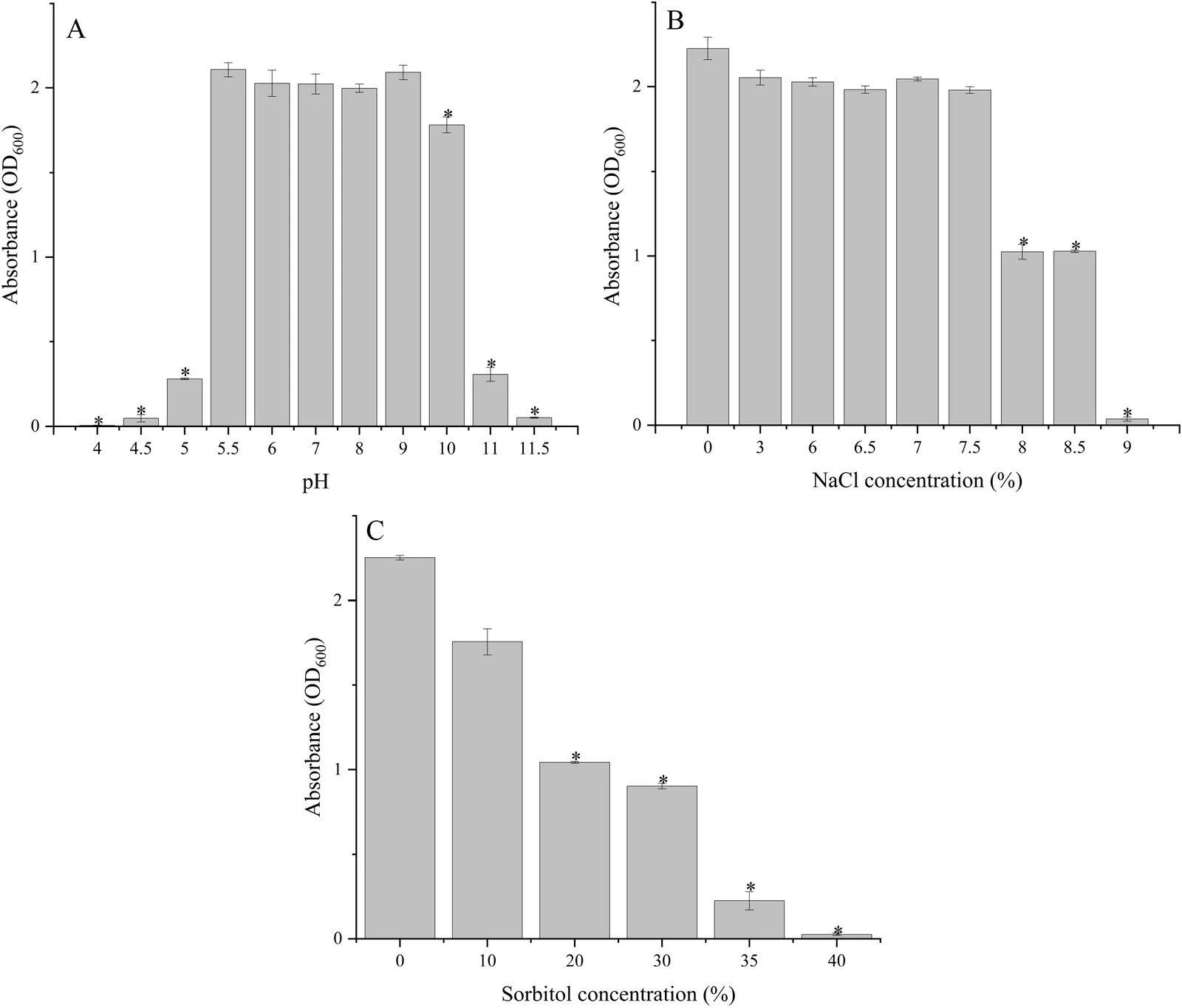
Growth of isolate ST3M-39a in nutrient broth under varying abiotic stress conditions. (A) Effect of initial pH (4–10). (B) Effect of increasing sodium chloride (NaCl) concentration (0-10% w/v). (C) Effect of sorbitol-induced osmotic stress (0-40% w/v). Data represent mean growth ± standard error (SE, n = 3). * indicate values significantly lower than the respective control condition (α = 0.05): pH 7.0 in panel (A), 0% NaCl in panel (B), and 0% sorbitol in panel (C).

#### 3.3.2 NaCl concentrations.

The isolate exhibited significant biological activity across a broad NaCl tolerance range of 0–8.5%, with measurable growth (absorbance > 1.0) observed up to 8.5% (Fig 3B). The assumption of homogeneity of variances was violated. Consequently, a Welch’s one-way ANOVA was performed, which revealed a highly significant effect of NaCl concentration on growth. Post hoc comparisons using the Games-Howell test showed that optimal growth occurred without NaCl (2.228 ± 0.066). Growth remained statistically comparable to control within the range of 3% to 7.5% NaCl, with no significant differences observed (all p > 0.05). While significant growth reduction occurred at 8%, 8.5%, and 9% compared to the control (all p < 0.05), measurable biological activity was still evident at 8.5% (1.027 ± 0.007). Therefore, the isolate maintains activity up to 8.5% NaCl but exhibits optimal growth across a wide, non-inhibitory range of 0–7.5%.

#### 3.3.3 Osmotic stress.

The isolate grew over a broad range of sorbitol concentrations (0%–35%), with absorbance values ranging from 0.225 to 2.253 (Fig 3C). The assumption of homogeneity of variances was violated. Therefore, Welch’s one-way ANOVA, which is robust to unequal variances, was used for the main analysis and revealed a highly significant effect of sorbitol concentration. Post hoc comparisons using the Games-Howell test showed that optimal growth, statistically equivalent to the sorbitol-free control (2.253 ± 0.014), was maintained up to 10% sorbitol (1.756 ± 0.078; p > 0.05). Significant yet measurable biological activity persisted up to 35% sorbitol, with absorbance values of 1.044 ± 0.005 at 20%, 0.902 ± 0.016 at 30%, and 0.225 ± 0.055 at 35%. Growth was fully inhibited at 40% (0.026 ± 0.006). Thus, the isolate exhibits an optimal growth range of 0–10% sorbitol and maintains biological activity under osmotic stress up to 35% sorbitol.

### 3.4 Phosphate solubilization under different conditions

#### 3.4.1 pH levels.

The isolate demonstrated significant phosphate solubilization capacity across various pH conditions (4.5, 7.0, and 8.5), with soluble phosphate concentrations ranging from 108.42 to 133.34 µg/mL (Fig 4A). The assumption of homogeneity of variance was confirmed by Levene’s test. A one-way ANOVA revealed a significant effect of initial pH on solubilization efficiency (p < 0.001, η² = 0.91), indicating that 91% of the variance in phosphate release was attributable to medium pH. Post hoc comparisons using Tukey’s HSD test showed that maximum phosphate release occurred at neutral pH (133.35 ± 3.23 µg/mL at pH 7.0). Solubilization was significantly reduced under both acidic and alkaline conditions, yielding 118.29 ± 1.73 µg/mL at pH 4.5 and 108.42 ± 1.30 µg/mL at pH 8.5 (both p < 0.05 versus pH 7.0). Following incubation, the final medium pH was 5.21 ± 0.01 (initial pH 4.5), 5.03 ± 0.01 (initial pH 7.0), and 5.32 ± 0.01 (initial pH 8.5). The most pronounced pH decrease occurred in the culture initially adjusted to neutral pH (Fig 4A).

**Fig 4:**
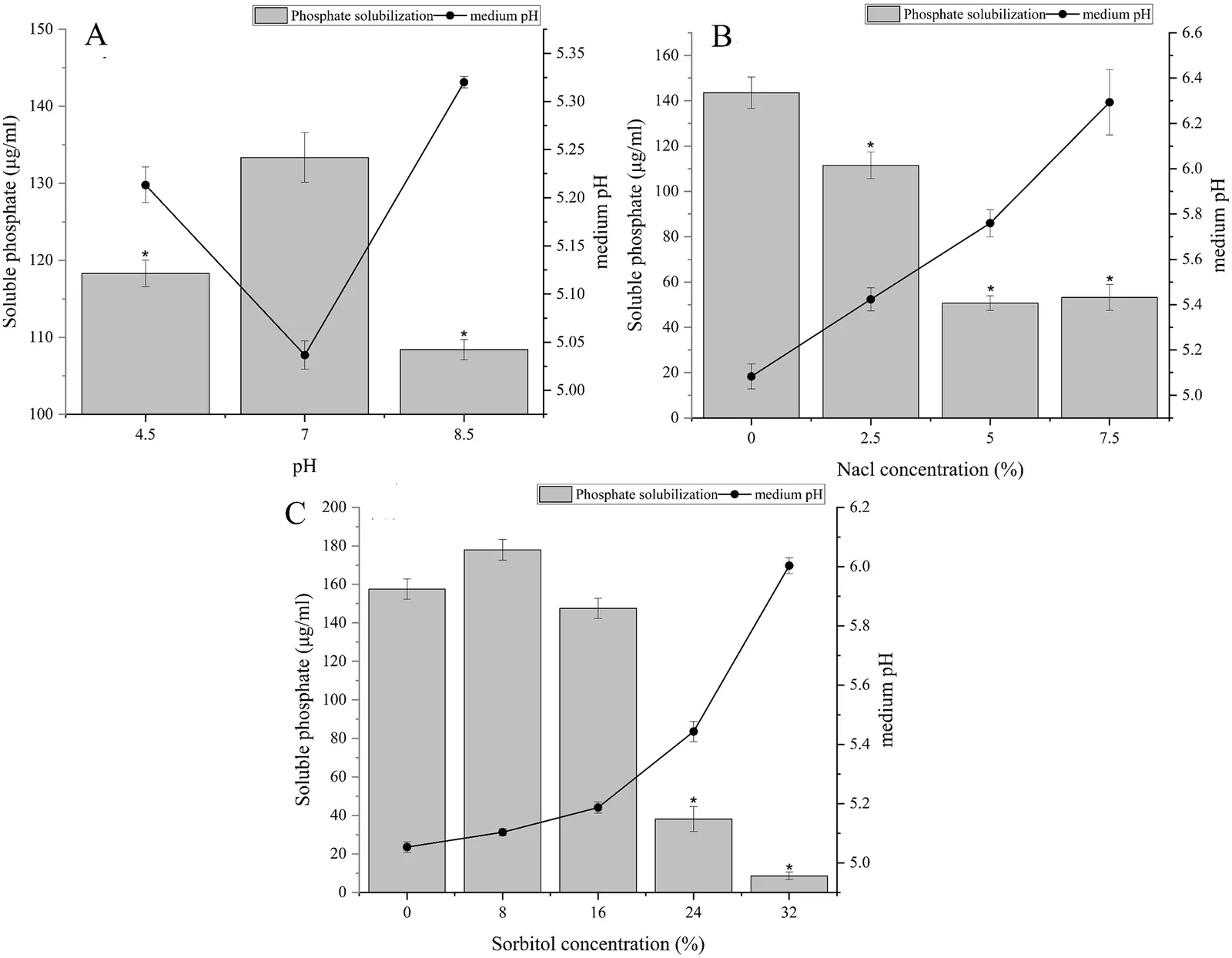
Phosphate solubilization by isolate ST3M-39a in NBRIP medium under varying abiotic stresses. (A) Effect of initial pH. (B) Effect of increasing NaCl concentration. (C) Effect of sorbitol-induced osmotic stress. Data represent mean ± SEs (n = 3). *Significantly lower than the respective control: pH 7 (A), 0% NaCl (B), 0% sorbitol (C); (α = 0.05). All assays used NBRIP medium with the specified stressor. Bars represent soluble phosphate (µg/mL); lines represent final pH.

#### 3.4.2 NaCl concentration.

The isolate exhibited different phosphate solubilization capacities across a range of NaCl concentrations (0–7.5%), releasing soluble phosphate concentrations ranging from 50.66 to 143.58 µg/mL (Fig 4B). The assumption of homogeneity of variance was confirmed by Levene’s test. A one-way ANOVA revealed a significant effect of NaCl concentration on phosphate solubilization (p < 0.001, η² = 0.96), indicating that approximately 96% of the variance in phosphate release was attributable to salinity. Post hoc comparisons using Tukey’s HSD test showed that maximum solubilization occurred in the absence of NaCl (143.58 ± 6.94 µg/mL). This activity was significantly inhibited by increasing salinity, with phosphate release decreasing to 111.47 ± 5.91 µg/mL at 2.5% NaCl and further decreasing to 50.66 ± 3.23 µg/mL at 5% NaCl and 53.21 ± 5.68 µg/mL at 7.5% NaCl (all p < 0.05 versus the 0% NaCl control). Similarly, the initial medium pH of 6.8 decreased during phosphate solubilization across all NaCl concentrations (Fig 4B). The most pronounced reduction occurred without NaCl (final pH 5.08 ± 0.05), with progressively smaller decreases observed at higher salt concentrations: pH 5.4 ± 0.05 (2.5% NaCl), pH 5.76 ± 0.06 (5% NaCl), and pH 6.29 ± 0.14 (7.5% NaCl).

#### 3.4.3 Osmotic stress.

The isolate exhibited varying phosphate solubilization capacities across different sorbitol concentrations (0–32%), releasing soluble phosphate concentrations ranging from 8.59 to 177.96 µg/mL (Fig 4C). The assumption of homogeneity of variance was confirmed by Levene’s test. A one-way ANOVA revealed a significant effect of sorbitol concentration on phosphate solubilization (p < 0.001, η² = 0.99), indicating that 99% of the variance in phosphate release was explained by osmotic stress. Post hoc comparisons using Tukey’s HSD test indicated that solubilization at 8% (177.96 ± 5.26 µg/mL) and 16% sorbitol (147.55 ± 5.17 µg/mL) remained statistically comparable to the 0% control (157.50 ± 5.26 µg/mL; both p > 0.05). In contrast, a significant reduction occurred at higher concentrations, decreasing to 38.15 ± 6.42 µg/mL at 24% and 8.59 ± 1.99 µg/mL at 32% sorbitol (both p < 0.05 versus the control). Similarly, the initial medium pH of 6.8 decreased during phosphate solubilization at all sorbitol concentrations (Fig 4C). Maximum acidification occurred without sorbitol (final pH 5.05 ± 0.02), with progressively smaller pH reductions observed at increasing concentrations: pH 5.10 ± 0.01 (8%), pH 5.18 ± 0.02 (16%), pH 5.44 ± 0.03 (24%), and pH 6.00 ± 0.02 (32%).

### 3.5 Other PGPR properties

#### 3.5.1 Zinc solubilization, EPS production, cellulase activity, protease activity, ammonia production, and biosurfactant production.

The isolate demonstrated a zinc solubilization ability, forming a clear halo zone on mineral salt media supplemented with zinc oxide (Fig 5A), with a solubilization efficiency of 152.19% ± 7.25%. Exopolysaccharide (EPS) production was 183.33 ± 14.53 µg/mL. Cellulase production was evident through the formation of a hydrolysis zone (Fig 5B), whereas protease activity was confirmed by the presence of a clearing zone (Fig 5C). Ammonia production was verified by a distinct color change from yellow to brown in the medium broth (Fig 5D). Additionally, biosurfactant production was verified through a positive drop collapse test (Fig 5E).

**Fig 5:**
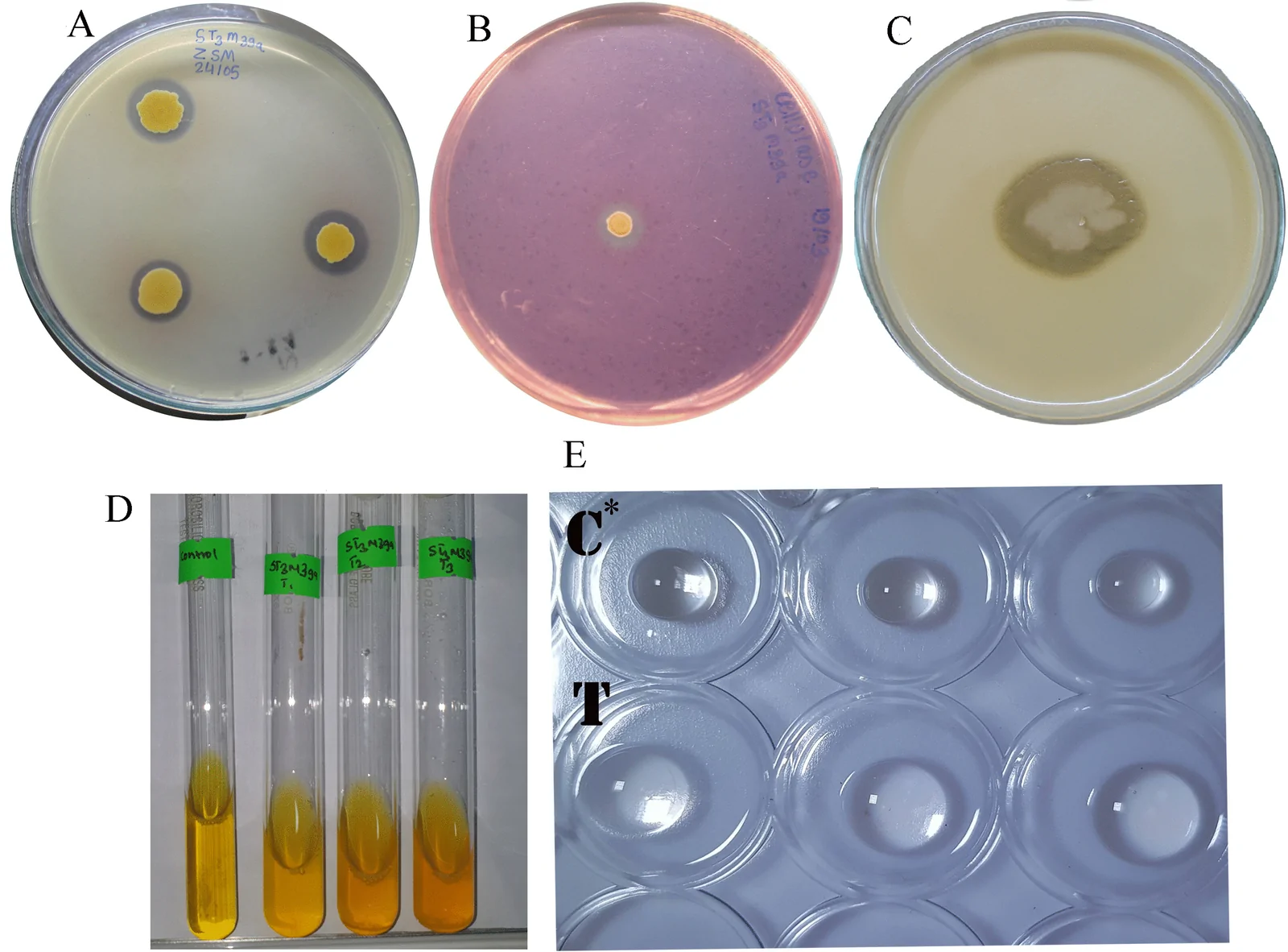
Multi-functional PGPR properties of isolate ST3M-39a. (A) Zinc solubilization on ZnO-supplemented mineral salt media (halo zone). (B) Cellulase activity on CMC agar (halo zone). (C) Protease activity on skim milk agar (halo zome). (D) Ammonia production. (E) Positive drop collapse test (biosurfactant production; C*: control, and T: test).

### 3.6 Safety assessment

ST3M-39a demonstrated no pathogenic or hemolytic activity as shown in Fig 6. Microscopic observation (Fig 6B and 6C) of bok choy leaves revealed no necrosis or lesions, and the strain showed no hemolysis on blood agar (Fig 6D). Statistical analysis of growth parameters via paired t-tests revealed that ST3M-39a treatment significantly increased leaf dimensions compared to controls. Leaf length was significantly greater in treated plants (mean = 19.008 ± 0.409 cm) than in controls (mean = 17.225 ± 0.324 cm) (p = 0.013, η² = 0.443), indicating that 44.3% of the variation in leaf length was due to treatment. Similarly, leaf breadth was significantly larger in the treated group (mean = 13.550 ± 0.339 cm) versus controls (mean = 11.817 ± 0.326 cm) (p = 0.007, η² = 0.498), indicating that 49.8% of the variation in leaf breadth was due to treatment. In contrast, the difference in root length between treated (mean = 11.675 ± 0.699 cm) and control plants (mean = 10.317 ± 0.422 cm) was not statistically significant (p = 0.064, η² = 0.278). However, the effect size suggested that the treatment accounted for 27.8% of the variance in root length, indicating a potentially meaningful biological difference that may become significant with a larger sample size.

**Fig 6:**
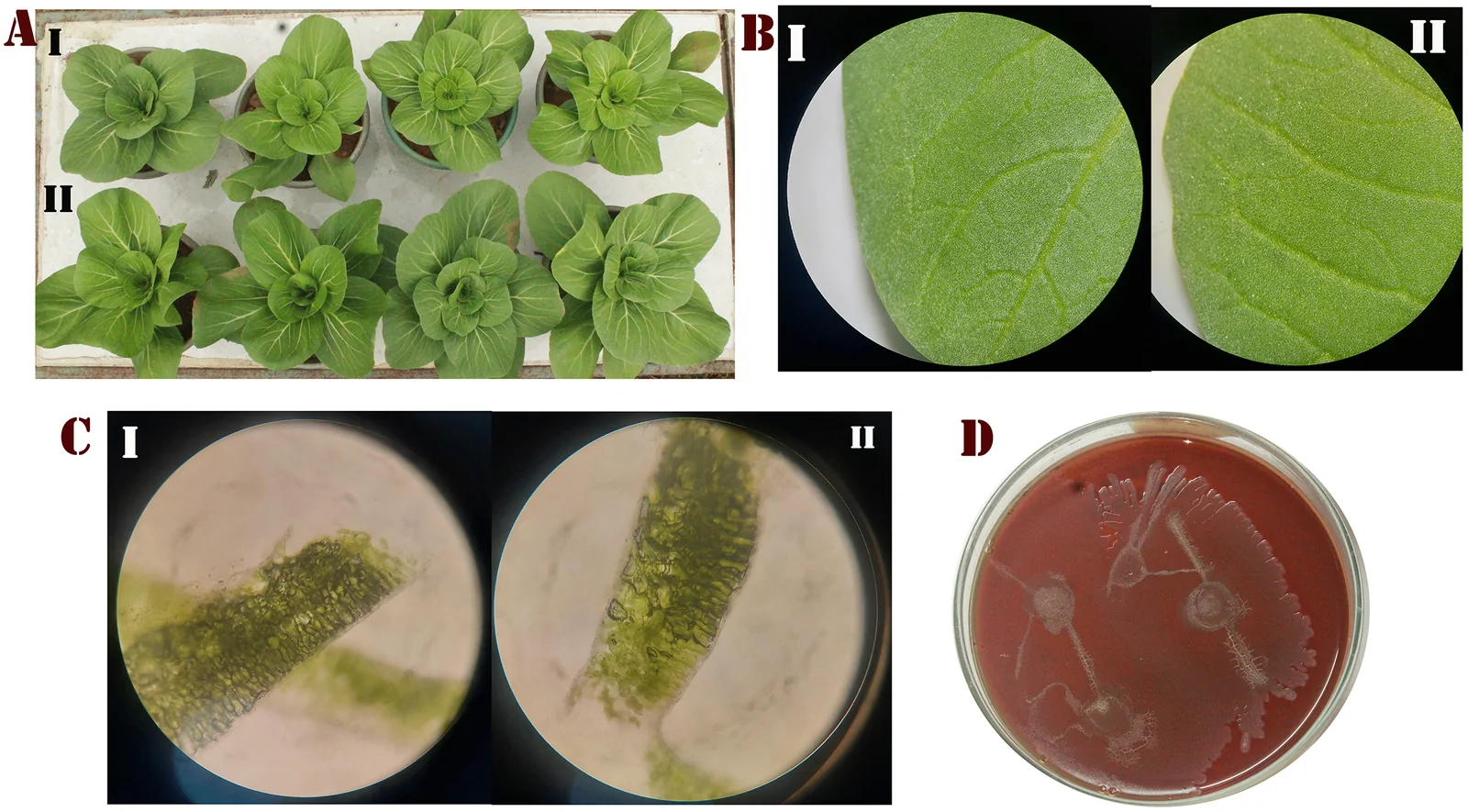
Safety assessment of isolate ST3M-39a. (A) Bok choy plants two weeks after foliar spray: Row I – control (nutrient broth); Row II - ST3M‑39a-treated. (B) Stereomicroscopic view (8X) of leaves: I – control, II – treated (no lesions/necrosis). (C) Light microscopic view (100X) of leaf mesophyll cells: I – control, II – treated (intact cell structure). (D) Growth on sheep blood agar after 24 h at 37°C showing γ‑hemolysis (non-hemolytic).

### 3.7 Greenhouse experiment

As shown in the radar plot (Fig 7), compared with the negative control samples, the test samples presented notable advantages in terms of aerial growth characteristics and yield, along with improved root development. On the other hand, compared with the test samples, the positive control samples presented enhanced growth performance with superior results in terms of chlorophyll content, root length, spike development, biomass accumulation, and yield. However, the test sample presented greater shoot length than did the positive control. Compared with negative control, both the test samples and the positive control significantly improved growth across all the measured parameters.

**Fig 7:**
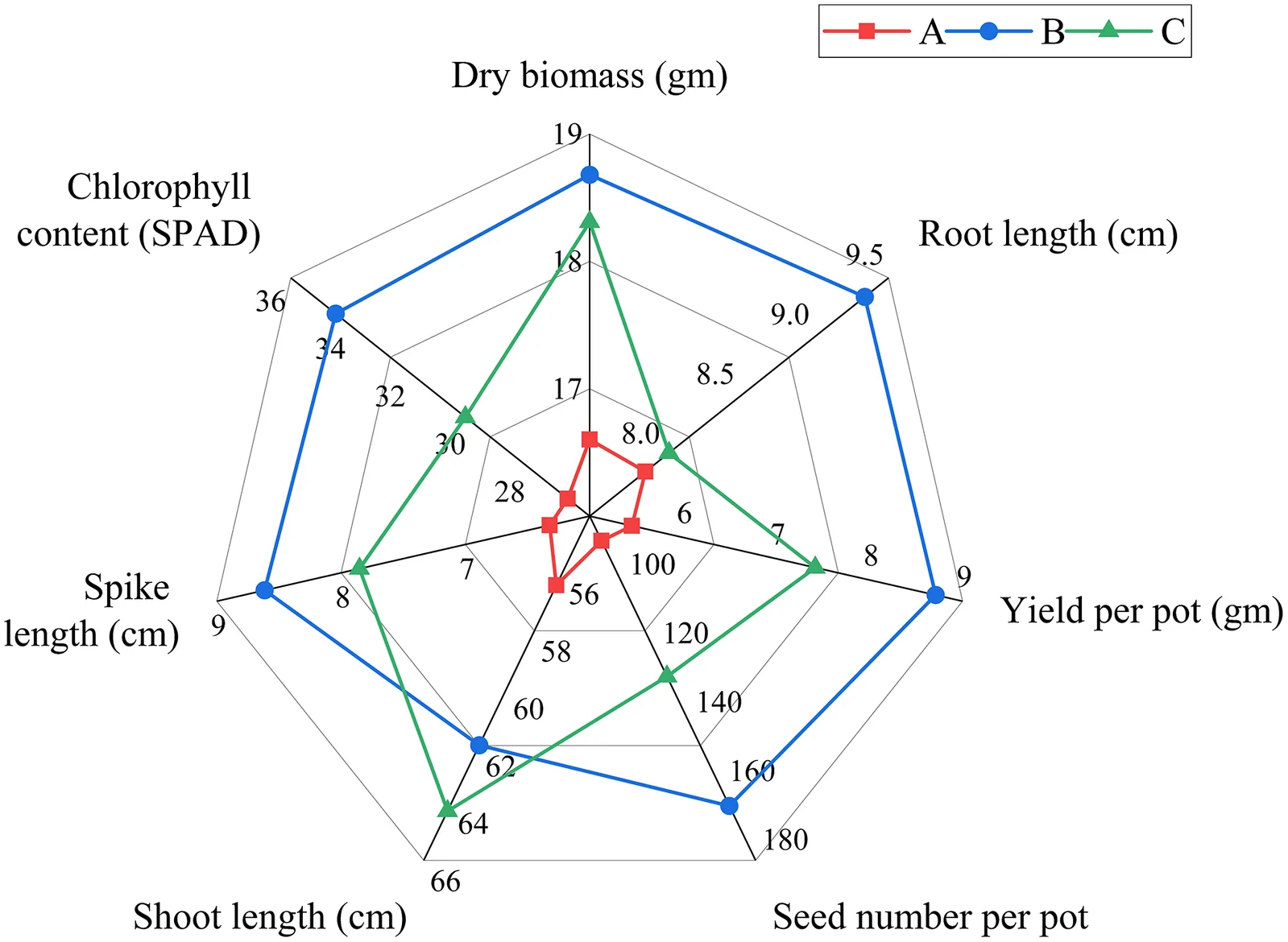
Radar plot of greenhouse trial outcomes. (A): negative control (no phosphate), (B): DAP-supplemented positive control, (C): ST3M-39a inoculated test samples. Each axis corresponds to a measured growth or yield parameter. Radical values are normalized means, allowing visual comparison of treatment performance across multiple traits.

#### 3.7.1 Effects on yield.

**3.7.1.1 Chlorophyll content:** Chlorophyll content analysis revealed significant variations among the treatment groups (Fig 8I). The assumption of homogeneity of variance was confirmed by Levene’s test. A one-way ANOVA revealed a large and statistically significant effect of treatment on chlorophyll levels (p < 0.001, η² = 0.70), indicating that 70% of the total variance in chlorophyll content was attributable to the treatment. Post hoc comparisons using Tukey’s HSD test showed that the positive control presented the highest mean chlorophyll content (34.49 ± 0.57 SPAD), representing a 14.4% increase over that of the test samples (30.15 ± 0.40 SPAD) and a 29.1% increase over the negative control (26.73 ± 0.67 SPAD; both p < 0.05). The test samples also maintained chlorophyll levels significantly higher (by 12.8%) than those of the negative control (p < 0.05).

**Fig 8:**
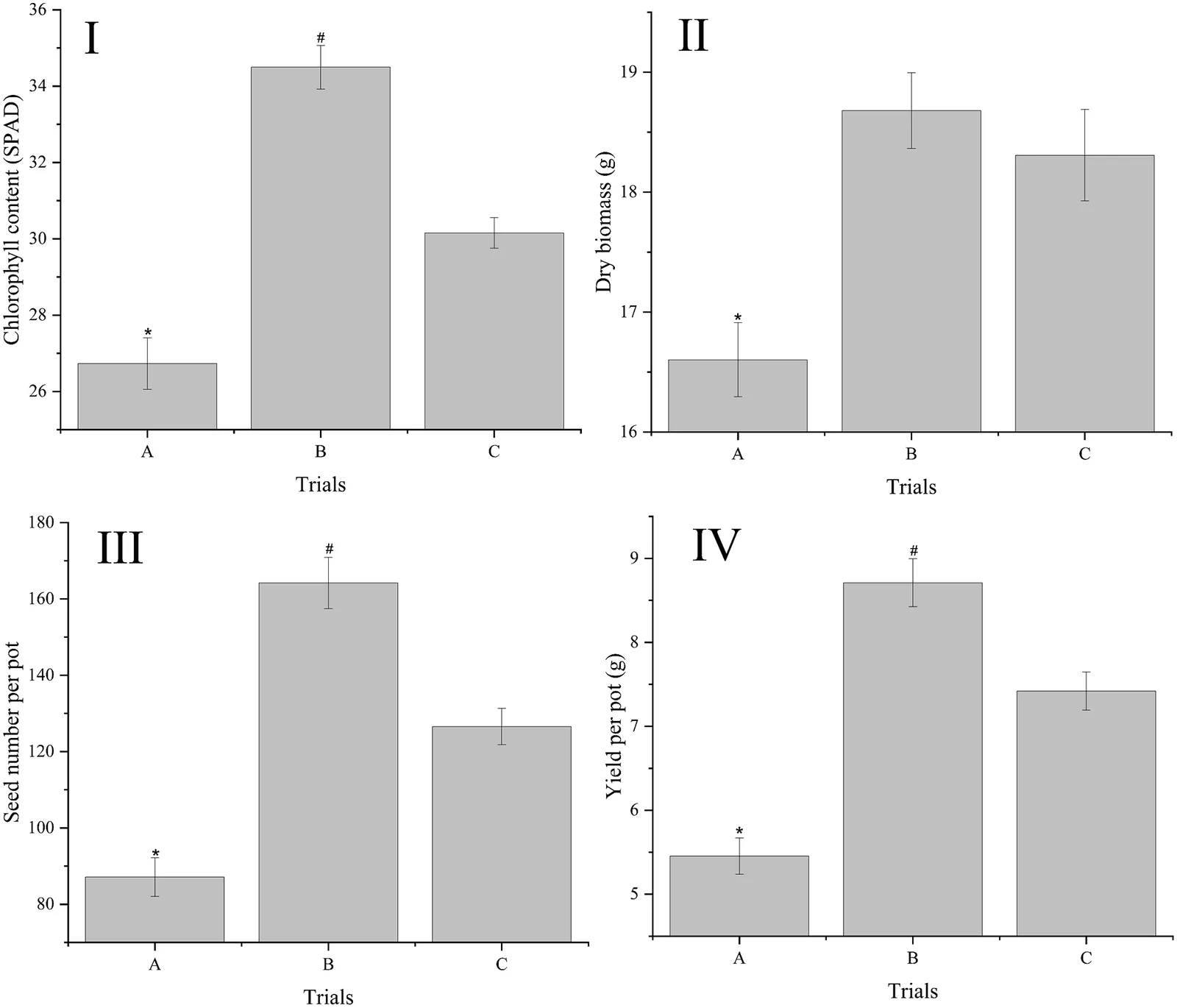
Yield parameters of wheat plants under different treatments. (I) Leaf chlorophyll content, (II) dry biomass, (III) seed count, and (IV) yield per pot. Treatments: (A) negative control, (B) DAP-supplemented positive control, (C) ST3M-39a inoculated test samples. The data represent means ± SEs (n = 15). *Significantly lower than treatment C (α = 0.05). ^#^Significantly higher than treatment C (α = 0.05).

**3.7.1.2 Dry biomass per pot:** The dry biomass of wheat varied significantly across treatments, ranging from 16.60 to 18.68 g (Fig 8II). The assumption of homogeneity of variance was confirmed by Levene’s test. A one-way ANOVA revealed a statistically significant and moderate effect of treatment on dry biomass (p < 0.001, η² = 0.34), indicating that approximately 34% of the total variance was explained by the treatment. Post hoc comparisons using Tukey’s HSD test showed that the test samples presented a mean dry biomass of 18.30 ± 0.38 g, representing a significant 10.2% increase over the negative control (16.60 ± 0.31 g; p < 0.05). While the positive control resulted in the greatest biomass (18.68 ± 0.31 g), it was not statistically different from the test samples (p > 0.05). However, the biomass of the positive control was significantly greater than that of the negative control (p < 0.05).

**3.7.1.3 Seed number per pot:** Seed production in wheat plants was significantly influenced by treatment (Fig 8III). The assumption of homogeneity of variance was confirmed. A one-way ANOVA revealed a large and statistically significant effect (p < 0.001, η² = 0.69), indicating that approximately 69% of the total variance in seed yield was attributable to the treatment factor. Post hoc analysis using Tukey’s HSD test delineated the group differences: the positive control yielded the highest seed count (164.2 ± 6.73), which was significantly greater than both the test samples (126.6 ± 4.73) and the negative control (87.13 ± 5.08) (all p < 0.05). Critically, the test samples themselves produced 45.3% more seeds than the negative control (p < 0.05), demonstrating a substantial, though intermediate, growth-promoting effect.

**3.7.1.4 Grain yield per pot:** Yield per pot varied significantly among treatments (Fig 8IV). The assumption of homogeneity of variance was confirmed by Levene’s test. A one-way ANOVA revealed a large and significant effect of treatment on yield (p < 0.001, η² = 0.68), indicating that approximately 68% of the total variance in yield was attributable to the treatment. Post hoc comparisons using Tukey’s HSD test showed a clear performance gradient: the positive control yielded the highest (8.71 ± 0.28 g), significantly outperforming both the test samples (7.42 ± 0.22 g; p < 0.05) and the negative control (5.45 ± 0.22 g; p < 0.05). Critically, the test samples themselves produced 36.1% greater yield than the negative control (p < 0.05), demonstrating substantial growth promotion, albeit intermediate relative to the positive control (85.2% of its yield potential).

#### 3.7.2 Effects on plant growth.

**3.7.2.1 Spike length:** The spike length of wheat plants varied significantly among treatments (Fig 9I). The assumption of homogeneity of variance was confirmed by Levene’s test. A one-way ANOVA revealed a large and significant effect of treatment on spike length (p < 0.001, η² = 0.65), indicating that approximately 65% of the total variance in spike length was attributable to the treatment. Post hoc comparisons using Tukey’s HSD test delineated the treatment effects: the positive control produced the longest spikes (8.61 ± 0.15 cm), which were significantly longer than both the test samples (7.85 ± 0.18 cm; p < 0.05) and the negative control (6.32 ± 0.21 cm; p < 0.05), representing a 36.2% increase over the negative control. Critically, the test samples themselves produced spikes 24.2% longer than the negative control (p < 0.05), while remaining 8.8% shorter than the positive control (p < 0.05).

**Fig 9:**
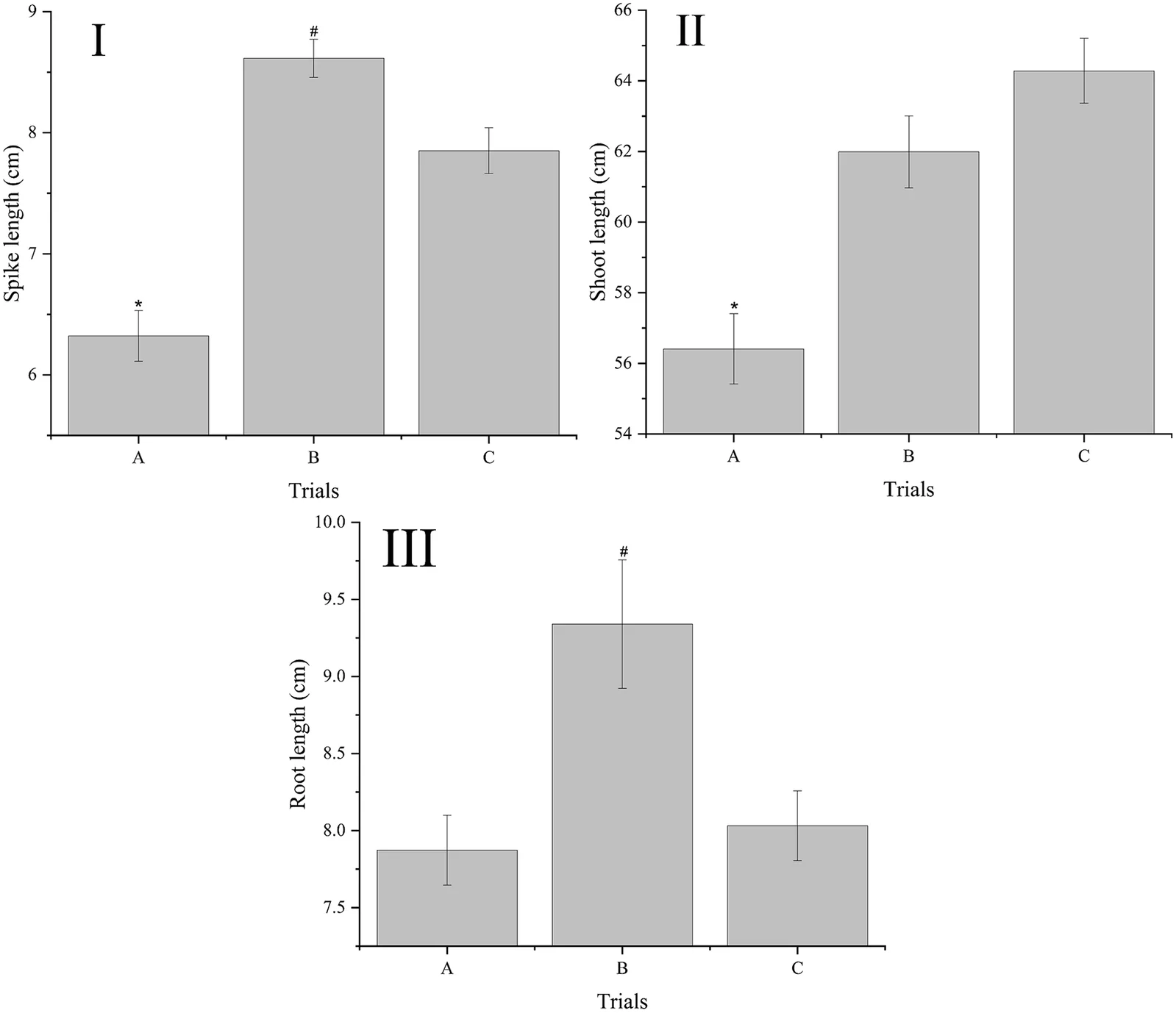
Growth parameters of wheat plants under different treatments. (I) Spike length, (II) shoot length, and (III) root length. Treatments: (A) negative control, (B) DAP-supplemented positive control, (C) ST3M-39a inoculated test samples. The data represent means ± SEs (n = 15). *Significantly lower than treatment C (α = 0.05). ^#^Significantly higher than treatment C (α = 0.05).

**3.7.2.2 Shoot length:** The shoot length measurements revealed significant differences among the treatments (Fig 9II). The assumption of homogeneity of variance was confirmed by Levene’s test. A one-way ANOVA revealed a significant effect of treatment on shoot elongation (p < 0.001, η² = 0.45), indicating that 45% of the total variance in shoot length was attributable to the treatment. Post hoc comparisons using Tukey’s HSD test showed that the test samples produced greater shoot elongation (64.28 ± 0.92 cm), which significantly exceeded that of the negative control (56.40 ± 0.99 cm) by 14.0% (p < 0.05). Notably, the test samples were statistically comparable to the positive control (61.98 ± 1.01 cm; p > 0.05), despite a nominal 3.7% difference. The positive control itself resulted in 9.9% greater shoot length than the negative control (p < 0.05).

**3.7.2.3 Root length:** The root length measurements varied among the treatments (Fig 9III), with values ranging from 7.87 to 9.34 cm. The assumption of homogeneity of variance was violated. Consequently, a Welch’s one-way ANOVA was conducted, which revealed a significant effect of treatment on root development. Post hoc comparisons using the Games-Howell test showed that positive control resulted in significantly greater root length (9.34 ± 0.42 cm) than both the test samples (8.03 ± 0.22 cm; p < 0.05) and the negative control (7.87 ± 0.22 cm; p < 0.05). However, root lengths of the test samples and the negative control were statistically comparable (p > 0.05). This indicates that while the positive control significantly enhanced root growth, the test isolate did not promote root development beyond the level of the untreated control.

## 4. Discussion

*Burkholderia gladioli* ST3M-39a—isolated from the maize rhizosphere and taxonomically validated through integrated morphological, biochemical, and molecular analyses—exhibits robust plant growth-promoting traits with notable phosphate solubilization capacity, positioning it as a promising bioinoculant for sustainable agriculture. Phylogenetic clustering with reference *B. gladioli* strains and 16S rRNA sequence similarity (BLAST), coupled with phenotypic congruence (urease/oxidase production, growth at pH 8/7% NaCl, utilization of sucrose/citrate/L-arginine/L-lysine/L-threonine/L-tryptophan; data in S1 Table) absent in related species (*B. plantarii*, *B. glumae*, *B. perseverans*) [40,53,54], confirms its classification. Divergent catabolism of lactose/mannitol/pyruvate/L-cystein/glycine versus all compared species suggested strain-specific adaptations. The isolate was non-motile in SIM medium, consistent with the non-flagellate nature of *B. gladioli*, yet displayed spreading growth on agar surfaces, which may be attributed to alternative mechanisms such as swarming motility [51,55]. This places ST3M-39a within well-documented *Burkholderia* genus—a dominant phosphate-solubilizing taxon alongside *Pseudomonas* and *Bacillus* [56–58] —previously identified in maize rhizospheres [25,59–61]. Beyond phosphate solubilization, PSB enhances plant growth via phytohormones: auxins (root formation/cell elongation), cytokinins (cell division/senescence delay), and gibberellins (germination/stem elongation/flowering regulation) [62], consistent with reports of *B*. *gladioli* strains exhibiting ammonia production, siderophore activity, and significant plant growth promotion [25,63,64].

Phosphate solubilization by ST3M-39a was confirmed through halo zone formation on PVK and NBRIP agar, with quantitative efficiency (177.96 µg/mL in liquid NBRIP) exceeding *Serratia nematodiphila* RJ10 (86 µg/mL) [65] but lower than *Burkholderia* spp. (485–580 mg/L at 72 h) [66] and *B. gladioli* MEL01 (350 mg/L at 28 h) [31]. Its solubilization index (PSI = 1.27 PVK; PSI = 1.41 NBRIP) aligns with established media ranges [67–70], while near-equivalence to *Pseudomonas* sp. (183.2 mg/L, 72 h) [71] under shorter incubation suggests superior kinetics. These interspecies variations likely reflect divergent metabolic adaptations [58]. Concomitant acidification (pH 6.8 to 5.0) confirmed organic acid-mediated solubilization, primarily via malic, gluconic, oxalic, and citric acids [22,72,73], that chelates Fe³ ⁺ , Al³ ⁺ , Ca² ⁺ ions. Resulting stable metal-organic complexes prevents phosphate re-precipitation, enhancing phosphorus bioavailability [12]. Crucially, while abiotic stresses (pH fluctuations, salinity, osmotic stress) generally constrain microbial solubilization, ST3M-39a maintained robust growth and phosphate dissolution efficiency across all tested conditions.

ST3M-39a exhibited exceptional climate resilience, maintaining growth across extreme abiotic gradients (pH 5–11, 8.5% NaCl, 0.943 a_w_)—conditions inhibiting most microorganisms (only 16.7% of arid-environment isolates survive at a_w_ 0.897 [74,75]). Previous research indicates that certain plant growth-promoting rhizobacteria (PGPR) strains exhibit robust growth across broad abiotic stress ranges, including pH extremes (4.0–12.0), salinity (2.5–8% NaCl), and osmotic stress induced by 20% PEG 6000 [76]. ST3M-39a’s phosphate solubilization, while optimal at neutral pH without stressors (177.96 µg/mL), persisted under suboptimal conditions (pH 4.5–8.5, 8% NaCl, 0.950 a_w_), aligning with stress-adapted PGPR like *Priestia megaterium* [77]. This pattern also aligns with previous observations of reduced but maintained phosphate solubilization under osmotic stress [65]. This tolerance likely involves *Burkholderia*-characteristic adaptations: membrane remodeling, stress-protective molecules [78], EPS-mediated mechanisms. Secreted EPS mitigates salinity by chelating Na⁺ ions [79], while enhancing phosphate solubilization through dual pathways: (1) stabilizing dissolved phosphorus via carboxyl/phenolic hydroxyl groups that bind phosphate through ion exchange and hydrogen bonding [80], and (2) electrostatically repelling H⁺ from organic acids at mineral interfaces to regulate dissolution [81,82]. Critically, EPS sustains phosphorus nutrition at a_w_ 0.950—when most microbial activity ceases [83]—while protecting plant from stress damage [84]. This integrated stress adaptation and nutrient cycling capacity exceeds strains like *B. seminalis* 869T2 [85], positioning ST3M-39a as a premier bioinoculant candidate for climate-affected regions with saline, arid, or pH-extreme soils.

Strain ST3M-39a demonstrated a multifaceted PGPR profile, integrating phosphate/zinc solubilization, exopolysaccharide (EPS) production, protease/cellulase activity, and ammonia generation to collectively enhance plant nutrition and stress resilience. Its zinc oxide solubilization capacity matches established PGPR strains [42,86,87], Countering deficiency-induced impairments in carbohydrate metabolism, auxin synthesis, and antioxidant defense that cause chlorosis, stunted growth, and yield loss [88]. EPS production, the hallmark of *Burkholderia* species [47,89,90], enhances salt tolerance through improved osmoregulation and biomass accumulation [91]. Concurrently, protease and cellulase enzymes mediate dual functions: biocontrol via fungal cell wall degradation (proteolytic cleavage) and plant cell wall breakdown (hydrolysis of 1,4-β-D-glycosidic linkages), alongside nutrient mobilization [92,93]; Protease further reinforce stress tolerance through protein repair, metabolic regulation, and defense hormone modulation [94,95]. Additionally, microbial-derived ammonia provides bioavailable nitrogen, driving root/shoot elongation and biomass increase [96]. Finally, the strain also produced biosurfactants, which serve a dual function: aiding in surface translocation via swarming motility while also acting as bio‑emulsifiers to increase the bioavailability of soil‑bound nutrients and hydrophobic contaminants, thereby enhancing its value as a biofertilizer [97,98].

The pathogenicity and hemolysis assays confirmed that isolate ST3M-39a is non-phytotoxic, with no observable necrosis or lesions in bok choy leaves, and non-hemolytic on blood agar which is similar to previous study [51]. This demonstrated innocuity towards both plant and mammalian systems is a critical prerequisite for advancing microbial strains as biofertilizer agents [99]. To further de-risk its application and establish a complete biosafety profile, subsequent toxicological characterization is required. Furthermore, the isolate demonstrated plant growth-promoting potential, as treated plants exhibited significant increases in leaf length and leaf breadth, while root length showed no significant impact.

The pot experiment demonstrated that *B. gladioli* ST3M-39a significantly enhanced wheat growth and yield in acidic soil, where phosphorus is typically fixed—highlighting its specific utility in such constrained environments. This finding with the growth and yield parameters aligned with the established evidence that PSB improve plant growth, tissue phosphorus, and yield while reducing post-harvest soil organic matter and pH [100–104]. While inoculation achieved 82–90% of the fertilizer efficiency of DAP by leveraging multifuctional traits beyond phosphate solubilization [105], it did not match full chemical fertilization, reflecting the inherent kinetics of microbial nutrient mobilization [106]. These promising results are tempered by methodological constraints, including the use of sterile soil, a minimal inoculum dose, and reliance on proxy soil data, which limit direct mechanistic interpretation of phosphorus dynamics [107]. Consequently, future translation requires optimization of delivery systems, validation in non-sterile soils, and hybrid integration with reduced chemical phosphorus to bridge this efficacy gap [108]. With targeted development, strains like ST3M-39a offer a viable pathway toward sustainable fertilization, capable of maintaining yields while enhancing long-term soil health in climate-vulnerable agricultural systems [109].

While this study demonstrated the plant growth-promoting potential of *B. gladioli* ST3M-39a, several constraints temper the immediate translation of these findings. The use of sterilized soil and a minimal inoculum dose likely overestimate the strain’s field efficacy, and its colonization of wheat roots remains unverified. Interpretations of phosphorus dynamics are limited by reliance on poxy soil data rather than batch-specific analysis. Furthermore, the host-range efficiency of this maize isolate on wheat, and the safety profile typical of the *Burkholderia* genus, require explicit investigation. Subsequent research must employ non-sterile soils, direct nutrient and colonization tracking, and rigorous biosafety assessment to validate the agronomic utility of this strain.

## 5. Conclusion

*Burkholderia gladioli* ST3M-39a emerges as a transformative bioinoculant, demonstrating exceptional resilience to abiotic stresses, while maintaining robust phosphate solubilization (177.96 ± 5.26 µg/mL). Its non-pathogenic and multifunctional plant growth-promoting traits (zinc solubilization, EPS production, enzymatic activity, ammonia synthesis, and biosurfactant production) drove wheat yield and biomass to 85–92% of fertilizer-equivalent yields under controlled conditions. This convergence of climate adaptability and nutrient mobilization efficiency positions ST3M-39a as a scalable solution for revitalizing phosphorus-deficient marginal soils.

## Supporting information

S1 Fig Location of the sampling site: Dumre Beshi, Bharatpur-29, Chitwan, Nepal. Administrative boundaries of Nepal and Chitwan District were obtained from the Database of Global Administrative Areas (GADM version 4.1; https://gadm.org). The data were processed and visualized using QGIS version 3.28 (QGIS Development Team, 2023). The red marker (or equivalent symbol) indicates the precise sampling location at Dumre Beshi.
(PDF)

S1 Table Phenotypic characteristics of isolate ST3M-39a and comparison with closely related type strains. Closely related species were identified via 16S rRNA gene sequence analysis using BLAST. Phenotypic features of the test strain (ST3M-39a) were determined through laboratory assays conducted in this study. Phenotypic features of reference strains were obtained from published literature.
(DOCX)

S2 Table Statistical analysis of experimental data (analysis report). (DOCX)
