# Supplementary material for: Defying salinity, drought, and pH extremes: A multifunctional rhizobacterium, Burkholderia gladioli ST3M-39a, matches fertilizer efficacy in wheat via phosphate solubilization
Source: PLoS One. 2026 Oct 9;21(10):e0332355. doi: 10.1371/journal.pone.0332355 (PMC13652262; doi:10.1371/journal.pone.0332355)
Supplement: Supplementary file 1 [file pone.0332355.s001.pdf]

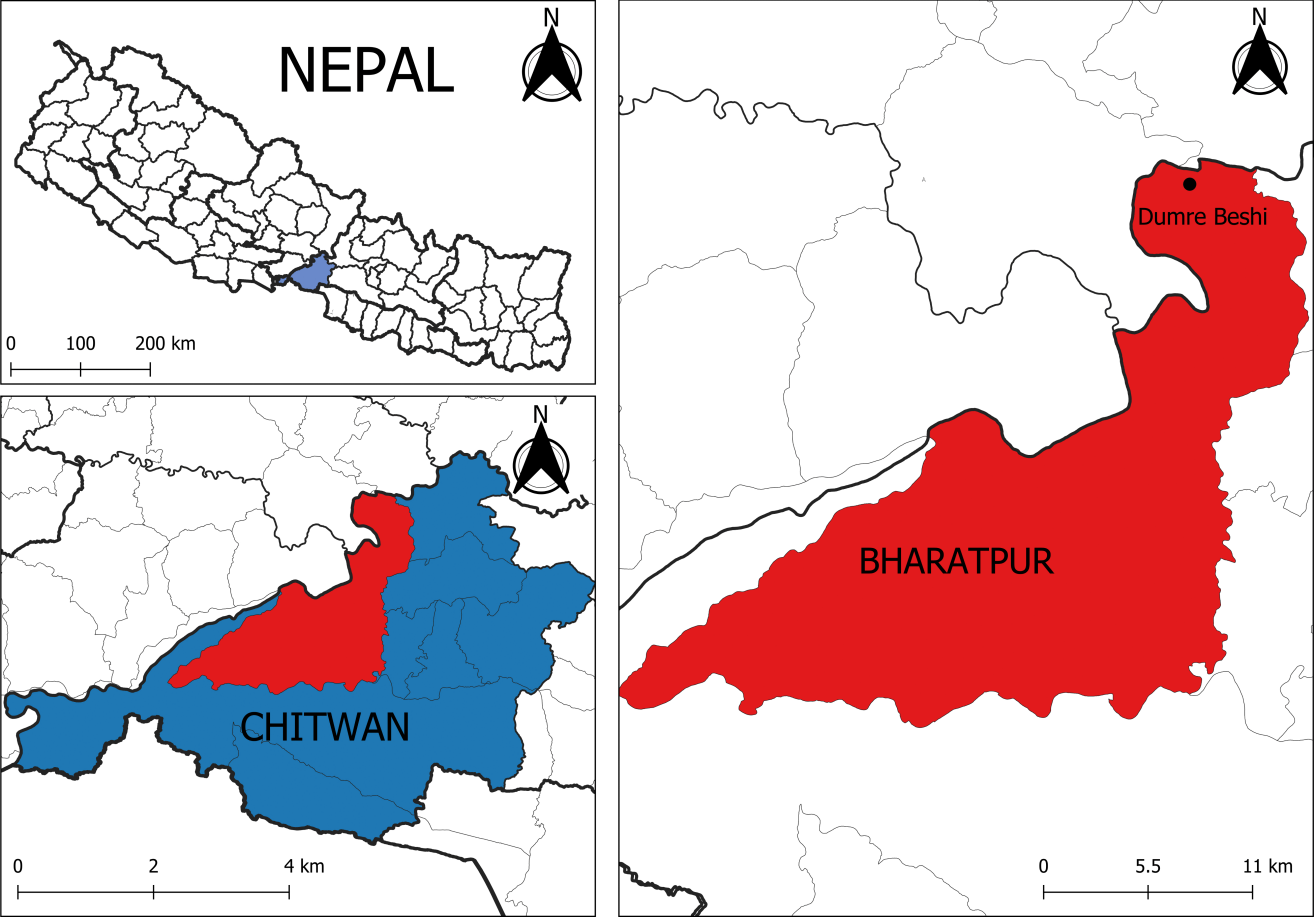
NEPAL
N
0
100
200 km
CHITWAN
N
0
2
4 km
BHARATPUR
Dumre Beshi
N
0
5.5
11 km
