# Supplementary material for: Defying salinity, drought, and pH extremes: A multifunctional rhizobacterium, Burkholderia gladioli ST3M-39a, matches fertilizer efficacy in wheat via phosphate solubilization
Source: PLoS One. 2026 Oct 9;21(10):e0332355. doi: 10.1371/journal.pone.0332355 (PMC13652262; doi:10.1371/journal.pone.0332355)
Supplement: Supplementary file 2 [file pone.0332355.s007.pdf]

Date: 2026/09/19

To,
The Editor
PLOS ONE

Subject: Revised Manuscript Submission - PONE-D-25-47295R2

Title: *Defying Salinity, Drought, and pH Extremes: A Multifunctional Rhizobacterium, Burkholderia gladioli ST3M-39a, Matches Fertilizer Efficacy in Wheat via Phosphate Solubilization*

Dear Editor,

Please find enclosed our revised manuscript (Manuscript ID: PONE-D-25-47295R2), which we are resubmitting for consideration for publication in PLOS ONE.

We are grateful to the editor and reviewer for the thoughtful and constructive feedback, which has greatly strengthened our manuscript. We have carefully addressed all comments and concerns in full. Key revisions include:

1. *2.1 protocol availability: Line No. 91 The protocols used in this study are available in protocols.io with DOI: dx.doi.org/10.17504/protocols.io.yxmvm17j6v3p/v1, please mention the name of the study to see the protocol availability:* We have mentioned the name of the study in protocol availability section as suggested.
2. *2.2 Materials and equipment: replaced with Chemical and Equipment:* As suggested, the heading has been changed to “Chemical and Equipment”.
3. *Figure 3 add the absorbance OD600 in x-axis:* We have corrected the error and have added OD_{600} to the x-axis.
4. *Figure 2 & 4 appears blurry, please use a clearer, high-resolution version:* We have uploaded the clearer, high-resolution versions of these figures.

All changes in the manuscript are highlighted for your convenience. We hope the revised manuscript is now suitable for publication in PLOS ONE. Thank you for your efforts in improving this manuscript.

With Best Regards,

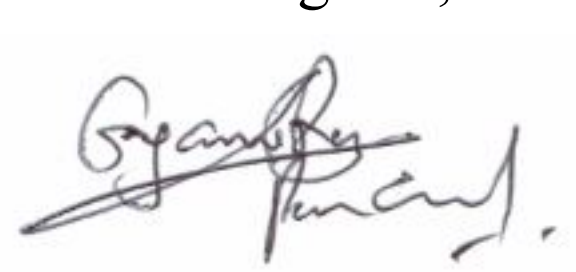

Gyanu Raj Pandey
Corresponding Author
Shubham Biotech Nepal Private Limited
Email: gyanupandey9@gmail.com
Phone number: +9779860470938
