# Supplementary material for: Defying salinity, drought, and pH extremes: A multifunctional rhizobacterium, Burkholderia gladioli ST3M-39a, matches fertilizer efficacy in wheat via phosphate solubilization
Source: PLoS One. 2026 Oct 9;21(10):e0332355. doi: 10.1371/journal.pone.0332355 (PMC13652262; doi:10.1371/journal.pone.0332355)
Supplement: Supplementary file 3 [file pone.0332355.s002.docx]

**S1 Table**: **Phenotypic characteristics of isolate ST3M-39a and comparison with closely related type strains.** Closely related species were identified via 16S rRNA gene sequence analysis using BLAST. Phenotypic features of the test strain (ST3M-39a) were determined through laboratory assays conducted in this study. Phenotypic features of reference strains were obtained from published literature.

| **Test** | **ST3M-39a** | ***B. gladioli*** (1) | ***B. perseverans*** (1,2) | ***B. glumae*** (1,3) | ***B. plantarii*** (1,3) |
| --- | --- | --- | --- | --- | --- |
| **Colony morphology** |  |  |  |  |  |
| Colony form | Circular |  | Round |  |  |
| Elevation | Raised |  | Low convex |  |  |
| Margin | Entire |  |  |  |  |
| Texture | Smooth |  | Smooth |  |  |
| Opacity | Opaque |  |  |  |  |
| Surface appearance | Glistening |  | Translucent |  |  |
| Chromogenesis | Creamy White |  |  |  |  |
| Diameter(mm) | 1.5 mm |  | 1mm |  |  |
| Pigmentation | - |  |  |  |  |
| **Microscopy** | gram-negative rods | gram-negative, non-spore forming rods | gram-negative,  non-spore forming rods |  |  |
| **VP Test** | - |  |  |  |  |
| **Indole Production** | - |  |  |  |  |
| **Motility (SIM media)** |  |  |  |  |  |
| **Hydrogen Sulphide Production** | - |  |  |  |  |
| **Production of:** |  |  |  |  |  |
| C4 lipase | + | + | + | v- | + |
| Amylase | - | v | v+ | v | v |
| Gelatinase | + | v+ | + | + | + |
| Protease | + | v+ | v+ | v- | + |
| Cellulase | + |  |  |  |  |
| Urease | + |  | - |  |  |
| **Growth in LB media** | + |  |  |  |  |
| **Growth in MacConkey Agar** | Pink colonies with no ppt around | - | + | - | - |
| **Oxidative in OF medium** | + |  |  |  |  |
| **Growth at** |  |  |  |  |  |
| 37°C | + |  | + |  |  |
| 40°C | + | + | + | + | + |
| 45°C | - |  |  |  |  |
| pH 8 | + | + | - | + | v- |
| 7% NaCl | + | + | - | - | - |
| **Catalase** | + |  | + |  |  |
| **Oxidase** | - | v | + |  |  |
| **Utilization of:** |  |  |  |  |  |
| Glucose | + | + | + |  |  |
| Sucrose | + | + |  | - | ± |
| Fructose | + | + |  | + | + |
| Maltose | - | - | - | - | - |
| Xylose | + | + |  | + | + |
| Galactose | + |  |  |  |  |
| Lactose | + | - | - | - | - |
| Mannitol | - | + | + |  |  |
| Melezitose | + |  |  |  |  |
| Sorbitol | + | + |  | + | + |
| Ethanol | + | + |  |  |  |
| Propanol | + | + |  |  |  |
| N-hexadecane | + |  |  |  |  |
| Sodium-K-Tartarate | + | + |  |  |  |
| Citrate | + | + | - | + | + |
| Pyruvate | - | + |  |  |  |
| L-Arginine | + | + | - | + | v |
| L-Cysteine | - | + |  | + | + |
| Glycine | + | - |  | - | - |
| L-Histidine | + | + |  | + | + |
| L-Lysine | + | + |  | - | - |
| L-Ornithine | - | + |  | - | - |
| L-Phenylalanine | + | + |  | + | + |
| L-Threonine | + | + |  | + | - |
| L-Tryptophan | + | + |  | + | - |
| L-Glutamine | + | + |  | + | + |
| **Resistant to antibiotics** | AMP, P, CTX, VA, RIF, CXM, CB |  |  |  |  |
| **Sensitive to antibiotics** | CIP, GEN, MO, LE, AZM, E |  |  |  |  |

v – variable, AMP – ampicillin (10 mcg); P – penicillin (1 unit); CTX – cefotaxime (30 mcg); VA – vancomycin (5 mcg); RIF – rifampicin (5 mcg); CXM – cefuroxime (30 mcg); CB – carbenicillin (100 mcg); CIP – ciprofloxacin (5 mcg); GEN – gentamycin (10 mcg); MO – moxifloxacin (5 mcg); LE – levofloxacin (5 mcg); AZM – azithromycin (15 mcg); E – erythromycin (15 mcg).

^1^ Brenner DJ, Krieg NR, Garrity GM, Staley JTTA-TT-. Bergey’s manual® of systematic bacteriology [Internet]. 2nd ed NV. Brenner DJ, Krieg NR, Staley JT, Garrity GM, editors. Boston, MA: Springer US; 2005. Available from: <http://link.springer.com/10.1007/0-387-28021-9>

^2^Andrade JP, de Souza HG, Ferreira LC, Cnockaert M, De Canck E, Wieme AD, eters C,
Gross E, De Souza JT, Marbach PAS, Góes-Neto A, Vandamme P. *Burkholderia perseverans* sp. nov., a bacterium isolated from the Restinga ecosystem, is a producer of volatile and diffusible compounds that inhibit plant pathogens. Braz J Microbiol [Internet]. 2021 Dec 21;52(4):2145–52. Available from: <https://doi.org/10.1007/s42770-021-00560-w>

^3^ Viallard V, Poirier I, Cournoyer B, Haurat J, Wiebkin S, Ophel-Keller K, Balandreau J. *Burkholderia graminis* sp. nov., a rhizospheric *Burkholderia* species, and reassessment of [*Pseudomonas*] *phenazinium*, [*Pseudomonas*] *pyrrocinia* and [*Pseudomonas*] *glathei* as *Burkholderia*. Int J Syst Bacteriol [Internet]. 1998 Apr 1;48(2):549–63. Available from: https://www.microbiologyresearch.org/content/journal/ijsem/10.1099/00207713-48-2-549
