# Supplementary material for: Defying salinity, drought, and pH extremes: A multifunctional rhizobacterium, Burkholderia gladioli ST3M-39a, matches fertilizer efficacy in wheat via phosphate solubilization
Source: PLoS One. 2026 Oct 9;21(10):e0332355. doi: 10.1371/journal.pone.0332355 (PMC13652262; doi:10.1371/journal.pone.0332355)
Supplement: Supplementary file 4 [file pone.0332355.s003.docx]

**S2 Table: Statistical Analysis of experimental data (analysis report).**

| **Experiment/Dependent Variable** | **Section** | **Levene’s F** | **Levene’s P** | **Var. Equal?** | **Primary Test** | **F / t Statistics** | **df** | **p-Value** | **η²** | **Post Hoc** |  |
| --- | --- | --- | --- | --- | --- | --- | --- | --- | --- | --- | --- |
| **A. Phosphate Solubilization – Inoculum Dose Optimization** | | | | | | | | | | | |
| **Inoculum Dose – Soluble phosphate released** | 3.1 | F (4,10) = 1.32 | 0.326 | Yes | One-way ANOVA | F (4,10) = 28.47 | 4, 10 | < 0.001 | — | Tukey's HSD |  |
| **B. Bacterial Growth** | | | | | | | | | | | |
| **Effect of pH** | 3.3.1 | F (10,22) = 4.17 | 0.0025 | No | Welch's ANOVA | F (10,8.33) = 1025.66 | 10, 8.33 | < 0.001 | — | Games-Howell |  |
| **Effect of NaCl concentration** | 3.3.2 | F (8,18) = 3.88 | 0.008 | No | Welch's ANOVA | F (8,7.33) = 1441.42 | 8, 7.33 | < 0.001 | — | Games-Howell |  |
| **Effect of Sorbitol concentration** | 3.3.3 | F (5,12) = 7.85 | 0.0017 | No | Welch's ANOVA | F (5,5.32) = 3518.36 | 5, 5.32 | < 0.001 | — | Games-Howell |  |
| **C. Phosphate Solubilization** | | | | | | | | | | | |
| **Effect of pH** | 3.4.1 | F (2,6) = 1.28 | 0.345 | Yes | One-way ANOVA | F (2,6) = 31.32 | 2, 6 | < 0.001 | 0.91 | Tukey's HSD |  |
| **Effect of NaCl concentration** | 3.4.2 | F (3,8) = 0.51 | 0.688 | Yes | One-way ANOVA | F (3,8) = 66.05 | 3, 8 | < 0.001 | 0.96 | Tukey's HSD |  |
| **Effect of Sorbitol concentration** | 3.4.3 | F (4,10) = 1.06 | 0.427 | Yes | One-way ANOVA | F (4,10) = 229.51 | 4, 10 | < 0.001 | 0.99 | Tukey's HSD |  |
| **D. Safety Assessment — Plant Pathogenicity (Bok Choy, n = 12 leaves per group)** | | | | | | | | | | |  |
| **ST3M-39a vs. control — bok choy leaf length (cm)** | 3.6 | — | — | — | Paired t-test | t (11) = 2.958 | 11 | = 0.013 | 0.443 | — |  |
| **ST3M-39a vs. control — bok choy leaf breadth (cm)** | 3.6 | — | — | — | Paired t-test | t (11) = 3.307 | 11 | = 0.007 | 0.498 | — |  |
| **ST3M-39a vs. control — bok choy root length (cm)** | 3.6 | — | — | — | Paired t-test | t (11) = 2.060 | 11 | = 0.064 **(ns)** | 0.278 | — |  |
| **E. Greenhouse Trial — Yield & Growth Parameters (n = 15 pots per treatment)** | | | | | | | | | | |  |
| **Treatment → chlorophyll content (SPAD)** | 3.7.1.1 | F (2,42) = 0.86 | 0.432 | Yes | One-way ANOVA | F (2,42) = 48.13 | 2, 42 | < 0.001 | 0.70 | Tukey's HSD |  |
| **Treatment → dry biomass per pot (g)** | 3.7.1.2 | F (2,42) = 0.16 | 0.853 | Yes | One-way ANOVA | F (2,42) = 10.80 | 2, 42 | < 0.001 | 0.34 | Tukey's HSD |  |
| **Treatment → seed count per pot** | 3.7.1.3 | F (2,42) = 1.62 | 0.209 | Yes | One-way ANOVA | F (2,42) = 47.58 | 2, 42 | < 0.001 | 0.69 | Tukey's HSD |  |
| **Treatment → grain yield per pot (g)** | 3.7.1.4 | F (2,42) = 2.85 | 0.069 | Yes | One-way ANOVA | F (2,42) = 44.82 | 2, 42 | < 0.001 | 0.68 | Tukey's HSD |  |
| **Treatment → spike length (cm)** | 3.7.2.1 | F (2,42) = 0.68 | 0.511 | Yes | One-way ANOVA | F (2,42) = 39.52 | 2, 42 | < 0.001 | 0.65 | Tukey's HSD |  |
| **Treatment → shoot length (cm)** | 3.7.2.2 | F (2,42) = 0.08 | 0.926 | Yes | One-way ANOVA | F (2,42) = 17.11 | 2, 42 | < 0.001 | 0.45 | Tukey's HSD |  |
| **Treatment → root length (cm)** | 3.7.2.3 | F (2,42) = 3.99 | 0.026 | No | Welch's ANOVA | F (2,26.74) = 4.81 | 2, 26.74 | = 0.016 | — | Games-Howell |  |

Notes: Var. Equal? = result of Levene's test for homogeneity of variance. When variance was unequal, Welch's ANOVA replaced standard one-way ANOVA, and Games-Howell replaced Tukey's HSD as post hoc. η² = eta-squared; — = not applicable. ns = not statistically significant (p > 0.05).
