# Supplementary material for: Defying salinity, drought, and pH extremes: A multifunctional rhizobacterium, Burkholderia gladioli ST3M-39a, matches fertilizer efficacy in wheat via phosphate solubilization
Source: PLoS One. 2026 Oct 9;21(10):e0332355. doi: 10.1371/journal.pone.0332355 (PMC13652262; doi:10.1371/journal.pone.0332355)
Supplement: Supplementary file 5 [file pone.0332355.s005.docx]

Date: 2026/01/01

To,
The Editor
PLOS ONE

**Subject: Revised Manuscript Submission - PONE-D-25-47295**

**Title:** *Defying Salinity, Drought, and pH Extremes: A Multifunctional Rhizobacterium, Burkholderia gladioli ST3M-39a, Matches Fertilizer Efficacy in Wheat via Phosphate Solubilization*

Dear Editor,

Please find enclosed our revised manuscript (Manuscript ID: PONE-D-25-47295), which we are resubmitting for consideration for publication in PLOS ONE.

We are grateful to the editor and reviewer for their thoughtful and constructive feedback, which has greatly strengthened our manuscript. We have carefully addressed all comments and concerns in full. Key revisions include:

1. *Enhanced Methodological and Statistical Rigor:* We have significantly expanded the Materials and Methods section to provide full details on soil characteristics, inoculation procedures, safety assessments, and statistical analysis.
2. *Transparent Reporting of Limitations:* A dedicated limitations and future perspective paragraph has been added to the discussion, providing a balanced interpretation of our findings.
3. *Compliance with Journal Policies:* We have updated the Funding and Competing Interests statements, clarified the copyright status of original figures, and added the required ethics statement for field sampling.
4. *Improved Data Presentation:* Figures and tables have been revised for clarity, with captions updated to accurately reflect the data sources and methodologies.

Below is a point-by-point summary of our responses to the editor’s and reviewers’ comments. All changes in the manuscript are highlighted for your convenience.

With Best Regards,
Gyanu Raj Pandey
Corresponding Author
Shubham Biotech Nepal Private Limited
Email: [gyanupandey9@gmail.com](mailto:gyanupandey9@gmail.com)
Phone number: +9779860470938

**Response to the Editor:**

**Comment 1: Protocol publication in “protocols.io”**

***Action:*** Thank you for encouraging us for making our protocol public. We have published the protocol in “protocol.io” with PROTOCOL-DOI “https://dx.doi.org/10.17504/protocols.io.yxmvm17j6v3p/v1”.

**Comment 2: Please ensure that your manuscript meets PLOS ONE's style requirements, including those for file naming.**

***Action:*** Manuscript is as per PLOS ONE’s style requirements.

**Comment 3: In your Methods section, please provide additional information regarding the permits you obtained for the work. Please ensure you have included the full name of the authority that approved the field site access and, if no permits were required, a brief statement explaining why.**

***Justification:*** Thank you for this request. As the soil sample were collected from private agricultural farmland with the landowner’s permission, and because the research did not involve endangered or protected species, no specific institutional or governmental permits were required for sample collection in this context. To clarify this for readers, we have added a statement to the **Materials and Method section (subsection 2.2).**

**Comment 4: Please include both an updated Funding Statement and Competing Interests Statement in your cover letter. We will change the online submission form on your behalf.**

***Action/Justification:*** Thank you for your guidance regarding the Funding Statement and Competing Interests declaration. We have updated the statements as:

*Funding Statement:* This work was supported by Shubham Biotech Nepal Private Limited. The funder provided support in the form of salaries for authors (Gyanu Raj Pandey, Sudip Silwal, and Asmita Shrestha) and research materials, but did not have any additional role in the study design, data collection and analysis, decision to publish, or preparation of the manuscript. The specific roles of these authors are articulated in the ‘Author Contributions’ section.

*Competing Interests:* The authors declare the following competing interests: Gyanu Raj Pandey and Asmita Shrestha are board members of Shubham Biotech Nepal Private Limited. Sudip Silwal is an employee of Shubham Biotech Nepal Private Limited. Shreejan Pokharel and Ramesh Acharya are employees of the Nepal Agricultural Research Council. The other author declare no competing interests. This does not alter our adherence to PLOS ONE policies on sharing data and materials.

**Comment 5: Thank you for uploading your study's underlying data set. Unfortunately, the repository you have noted in your Data Availability statement does not qualify as an acceptable data repository according to PLOS's standards.**

***Action:*** Data have been published in the Harvard Dataverse (<https://doi.org/10.7910/DVN/WV36TD>).

**Comment 6: Are there any copyright issues with figure 1?**

***Justification:*** Thank you for your note regarding the copyright status of Figure 1. We appreciate the journal’s strict adherence to licensing policies. We wish to clarify that **Figure 1 is an original map created entirely by the authors for this manuscript.** It was generated using the open-source geographic information system QGIS. The base geographic data (administrative boundaries for Nepal and the Chitwan district) were sourced from publicly available, non-proprietary databases. We obtained administrative boundaries for Nepal and Chitwan District from the Database of Global Administrative Areas (GADM version 4.1; <https://gadm.org>). Therefore, this figure is not subject to third-party copyright restrictions. As the creators, we grant PLOS ONE full permission to publish it under the Creative Commons Attribution License (CC BY 4.0).

**Responses to Reviewer Comments**

**Comment 1: The strain is a maize rhizosphere isolate, then why using it in wheat but not in maize for evaluating its growth-promotion?**

***Justification:*** "We thank the reviewer for this pertinent question. The primary rationale for testing this maize rhizosphere isolate on wheat was to assess its broader functional potential as a cross-crop plant growth-promoting rhizobacterium (PGPR). Many PGPR traits, particularly phosphate solubilization, are not strictly host-specific. This approach helps identify robust candidates with wide applicability. We acknowledge that evaluating its efficacy on its native host (maize) is a logical and important next step. We have amended the discussion to clarify this rationale and explicitly state that host-origin testing will be a focus of subsequent research."

**Comment 2: The greenhouse experiment is somewhat the most important part of this work. However, there is no details in section 2.8. No mention of the basic information of soil, e.g., the pH， Pi content, and salt content...**

***Action/Justification:*** We thank the reviewer for this essential critique. We have now amended **Section 2.9** to include detailed reference characteristics of the silty loam soil used, sourced from the same field site. The representative values are: pH 4.76, 2.65% organic matter, 163.35 mg kg⁻¹ P₂O₅, 55.31 mg kg^-1^ K_2_O, and 0.12% total nitrogen. We have added a sentence clarifying that these are reference values from the source site, provided to contextualize the experiment, as a full analysis of the specific batch was not conducted. Furthermore, in the **Discussion**, we now address the relevance of the soil's acidic pH for interpreting the phosphate-solubilizing activity of our isolate.

**Comment 3: In my opinion, if the authors want to claim the multifunction, such as mitigation of salinity, drought, and pH extremes, they should at least measures some representative indicators in wheat.**

***Justification:*** "We agree with the reviewer and have refined our language to avoid overstatement. The *in vitro* assays demonstrate the **bacterial strain's tolerance** to abiotic stresses, which is a desirable trait for a bioinoculant intended for challenging environments. We do not claim, based on the current data, that inoculation mitigates these stresses in plants.

**Comment 4: Also, if the conclusion is ST3M-39a matches fertilizer efficacy in wheat via phosphate solubilization, they need to measure the avaiblabe and total Pi contents in soils and plant tissues.**

***Justification:*** "This is a valuable and critical point. We acknowledge that measuring available P in the rhizosphere soil and total P in plant tissue at harvest would provide direct mechanistic evidence linking the strain's solubilization activity to plant P uptake. The absence of this data is a limitation of the current study. We have added a paragraph in the Discussion highlighting this limitation and stating that soil/plant P quantification is a prerequisite for advanced field trials."

**Comment 5: Line 199, the positive control is adding 90kg/ha diammonium phosphate, how this can be achieved in pot?**

***Justification:*** "The field application rate was converted to a pot-scale equivalent based on soil surface area. For a 9-inch (22.86 cm) diameter pot, the surface area is ~0.041 m². The field rate of 90 kg DAP/ha equals 9 g/m². Therefore, the amount per pot was 9 g/m² * 0.041 m² = **0.37 g DAP per pot**."

**Comment 6: Test samples was added 1 mL of phosphate-solubilizing bacterial inoculum, I am wondering how such a small volume was applied? And only once during the whole growing season of wheat?**

***Justification:*** "The 1 mL inoculum was applied as a soil drench near the base of seedlings at the time of thinning. This volume and single application were chosen for the sterile soil system to deliver a precise CFU count while minimizing soil disturbance. We recognize that for field application, a higher volume, carrier-based formulation, or seed treatment would be standard."

**Comment 7: I have no idea how table 1 was composed. How can they get phenotypic features and metabolic properties of these selected isolates based on BLAST?**

***Justification:*** "We apologize for the lack of clarity. Table 1 presents phenotypic and metabolic properties determined through laboratory assays. We have amended and clarified the caption accordingly."

**Comment 8: By the way, the colonization and replication of added bacterium in pots should be evaluated to confirm they can really have an effect.**

***Justification:*** "The reviewer raises a fundamental point. We did not monitor the colonization dynamics of ST3M-39a in the wheat rhizosphere in this experiment. Demonstrating successful root colonization is indeed crucial to confirm a direct cause-effect relationship. We have added a statement in the Limitations/Future Work section that 'future studies must employ molecular tools (e.g., qPCR with strain-specific primers or GFP-tagging) to quantify colonization and persistence of the inoculant in the rhizosphere.'"

**Comment 9: In addition, the potential safety issues should be tested or at least discussed before used as a bioinoculant.**

***Justification:*** " We sincerely thank the reviewer for raising this critical point regarding biosafety, which is essential for the responsible development of any microbial inoculant, particularly for strains within the Burkholderia genus. In direct response to this comment, we have performed two independent safety assessments and have integrated a detailed discussion of the results into the manuscript.

We performed plant pathogenicity assay on bok choy, microscopic examination revealed no necrosis, lesions, or cellular damage in treated leaves. Furthermore, treated plants showed **significant increases in leaf length and breadth** compared to controls, indicating a growth-promoting rather than pathogenic interaction. Also, the strain exhibited no zone of hemolysis on blood agar, suggesting it lacks this key virulence-associated trait."

**Comment 10: Line 357, the dry biomass of wheat varied significantly across treatments, ranging from 16.60 to 18.68 gm? What’s the unit “gm” standing for? I can not image the dry weight of a mature wheat seedlings is so light.**

***Action/Justification:*** "1. **Unit Correction:** 'gm' is a typographical error and has been corrected to the standard unit 'g' (grams) throughout the manuscript.

2. **Data Clarification:** The reported dry biomass (16.60-18.68 g) is for the **total shoot biomass per pot (5 plants) without spikes**, not per individual seedling. This has been clarified in the text. We have also double-checked our raw data and measurements to confirm their accuracy."

**Comment 11: Problems with statistical analysis**

***Action/Justification:* “**We sincerely thank reviewer for pointing out the major errors. All statistical analysis were performed again appropriately and rigorously.”
