# Supplementary material for: Defying salinity, drought, and pH extremes: A multifunctional rhizobacterium, Burkholderia gladioli ST3M-39a, matches fertilizer efficacy in wheat via phosphate solubilization
Source: PLoS One. 2026 Oct 9;21(10):e0332355. doi: 10.1371/journal.pone.0332355 (PMC13652262; doi:10.1371/journal.pone.0332355)
Supplement: Supplementary file 6 [file pone.0332355.s006.docx]

Date: 2026/05/30

To,
The Editor
PLOS ONE

**Subject: Revised Manuscript Submission - PONE-D-25-47295R1**

**Title:** *Defying Salinity, Drought, and pH Extremes: A Multifunctional Rhizobacterium, Burkholderia gladioli ST3M-39a, Matches Fertilizer Efficacy in Wheat via Phosphate Solubilization*

Dear Editor,

Please find enclosed our revised manuscript (Manuscript ID: PONE-D-25-47295R1), which we are resubmitting for consideration for publication in PLOS ONE.

We are grateful to the editor and reviewer for their thoughtful and constructive feedback, which has greatly strengthened our manuscript. We have carefully addressed all comments and concerns in full. Key revisions include:

1. *Enhanced captions and legends for figures and table:* We have improved captions and legends for figures and table. Captions are self-explanatory. Also, we have corrected figure names and captions as per journal’s requirements. For Fig 8 and Fig 9, we have used roman numbers for indicating multiple graphs, as capital alphabets were used for indicating treatment groups.
2. *Removed one figure and table from main text to supporting information:* As suggested by reviewer, we have moved Fig 1, and Table 1 to the supporting information.
3. *Removed some texts and statistical reporting as suggested by reviewer:* We have removed last paragraph of the conclusion. We have also removed some repetitive statistical reporting from the main text. We have also added S2 Table as supporting information for statistical reporting.

Below is a point-by-point summary of our responses to the editor’s and reviewers’ comments. All changes in the manuscript are highlighted for your convenience.

With Best Regards,
Gyanu Raj Pandey
Corresponding Author
Shubham Biotech Nepal Private Limited
Email: [gyanupandey9@gmail.com](mailto:gyanupandey9@gmail.com)
Phone number: +9779860470938

**Response to the Editor:**

**Comment: Please address the minor technical comments provided by the reviewer.**

***Action/Response:*** Thank you for managing the manuscript, we have carefully addressed the minor technical comments provided by the reviewer. We have improved the manuscript accordingly.

**Responses to Reviewer Comments**

**Reviewer #1:**

**Comment 1: I personally think figure 1 can be moved into supplementary information.**

***Action:*** We have moved figure 1 into supplementary information as Fig S1.

**Comment 2: Line 225, what’s the cultivar of Triticum aestivum?**

***Action:*** We thank reviewer for pointing out the missed information. We have included the cultivar in manuscript as *Triticum aestivum* ‘WK 1204’.

**Comment 3: Line 227, “each replicated 15 times”, what does this mean? How many technical repeats and how many biological replicates?**

***Action/Justification:*** This is valuable and critical observation; we thank reviewer for pointing this. We have had 15 biological replicates for pot experiment. We recorded and reported a single set of data for each 15 sets of pots for each treatment group. We have also included “biological replicates” in manuscript.

**Comment 4: Table 1 can also be moved into supplementary information.**

***Action:*** We have moved Table 1 into supplementary information as S1 Table.

**Comment 5 & 6: Figure 4 should be inserted under line 340 other than line 312. Similar suggestion for the location of figure 5.**

***Action:*** We thank reviewer for pointing out this error. We have addressed the issue and also, we have made some arrangements for other misplaced figures too.

**Comment 7 & 8: As the authors have already described the statistics of data in section 2.11, it seems quite distracting to show all F and P values in the results. In section 3.6, is it necessary to show (t(11) = xx, p = xx, η^2^ = xx)?**

***Action/Justification:*** This is indeed somehow distracting. We have addressed the issues in the manuscript. Also, we have added a supporting information S2 Table including statistical reports.

**Comment 9: The last paragraph can be deleted, as they are more like expectation by the authors other than conclusion.**

***Action:*** We have deleted the last paragraph.

**Reviewer #2: The manuscript titled “Defying Salinity, Drought, and pH Extremes: A Multifunctional Rhizobacterium, *Burkholderia gladioli* ST3M-39a, Matches Fertilizer Efficacy in Wheat via Phosphate Solubilization”, addresses an important and timely issue and has merit for publication. The paper is accepted subject to minor revisions aimed at improving overall quality and enhancing reader interest. Enhancing figures quality, consistent formatting, and self-explanatory titles, along with detailed statistical analysis in tables, will improve quality.**

***Action:*** We thank reviewer for this kind words and impactful suggestions. We have enhanced the figure quality, improved formatting and have provided self-explanatory titles. We have also added an S2 Table in supporting information that detailed the statistical analysis we have carried out.
