# Supplementary material for: Defying salinity, drought, and pH extremes: A multifunctional rhizobacterium, Burkholderia gladioli ST3M-39a, matches fertilizer efficacy in wheat via phosphate solubilization
Source: PLoS One. 2026 Oct 9;21(10):e0332355. doi: 10.1371/journal.pone.0332355 (PMC13652262; doi:10.1371/journal.pone.0332355)
Supplement: Peer review history

# Peer review: Decision Letter 0

**DOI:** 10.1371/journal.pone.0332355.r001

**Authors:** Abhijeet Kashyap

3 Oct 2025

Dear Dr. Pandey,

Thank you for submitting your manuscript to PLOS ONE. After careful consideration, we feel that it has merit but does not fully meet PLOS ONE’s publication criteria as it currently stands. Therefore, we invite you to submit a revised version of the manuscript that addresses the points raised during the review process.

Please submit your revised manuscript by Nov 17 2025 11:59PM. If you will need more time than this to complete your revisions, please reply to this message or contact the journal office at plosone@plos.org. When you're ready to submit your revision, log on to https://www.editorialmanager.com/pone/ and select the 'Submissions Needing Revision' folder to locate your manuscript file.

- A rebuttal letter that responds to each point raised by the academic editor and reviewer(s). You should upload this letter as a separate file labeled 'Response to Reviewers'.
- A marked-up copy of your manuscript that highlights changes made to the original version. You should upload this as a separate file labeled 'Revised Manuscript with Track Changes'.
- An unmarked version of your revised paper without tracked changes. You should upload this as a separate file labeled 'Manuscript'.

If you would like to make changes to your financial disclosure, please include your updated statement in your cover letter. Guidelines for resubmitting your figure files are available below the reviewer comments at the end of this letter.

If applicable, we recommend that you deposit your laboratory protocols in protocols.io to enhance the reproducibility of your results. Protocols.io assigns your protocol its own identifier (DOI) so that it can be cited independently in the future. For instructions see: https://journals.plos.org/plosone/s/submission-guidelines#loc-laboratory-protocols. Additionally, PLOS ONE offers an option for publishing peer-reviewed Lab Protocol articles, which describe protocols hosted on protocols.io. Read more information on sharing protocols at https://plos.org/protocols?utm_medium=editorial-email&utm_source=authorletters&utm_campaign=protocols.

We look forward to receiving your revised manuscript.

Kind regards,

Abhijeet Shankar Kashyap

Academic Editor

PLOS ONE

**Journal Requirements:**

1. When submitting your revision, we need you to address these additional requirements.

Please ensure that your manuscript meets PLOS ONE's style requirements, including those for file naming. The PLOS ONE style templates can be found at

https://journals.plos.org/plosone/s/file?id=wjVg/PLOSOne_formatting_sample_main_body.pdf and

https://journals.plos.org/plosone/s/file?id=ba62/PLOSOne_formatting_sample_title_authors_affiliations.pdf

2. In your Methods section, please provide additional information regarding the permits you obtained for the work. Please ensure you have included the full name of the authority that approved the field site access and, if no permits were required, a brief statement explaining why.

3. Thank you for stating the following in the Competing Interests section:

The authors declare the following financial interests/personal relationships which may be considered as potential competing interests:

Gyanu Raj Pandey reports administrative support and equipment, drugs, or supplies were provided by Shubham Biotech Nepal Private Limited. Gyanu Raj Pandey reports a relationship with Shubham Biotech Nepal Private Limited that includes: board membership. Sudip Silwal reports a relationship with Shubham Biotech Nepal Private Limited that includes: employment. Asmita Shrestha reports a relationship with Shubham Biotech Nepal Private Limited that includes: board membership. Shreejan Pokharel reports a relationship with Nepal Agricultural Research Council Biotechnology Unit that includes: employment. Ramesh Acharya reports a relationship with Nepal Agricultural Research Council Biotechnology Unit that includes: employment. If there are other authors, they declare that they have no known competing financial interests or personal relationships that could have appeared to influence the work reported in this paper.

We note that one or more of the authors are employed by a commercial company.

a. Please provide an amended Funding Statement declaring this commercial affiliation, as well as a statement regarding the Role of Funders in your study. If the funding organization did not play a role in the study design, data collection and analysis, decision to publish, or preparation of the manuscript and only provided financial support in the form of authors' salaries and/or research materials, please review your statements relating to the author contributions, and ensure you have specifically and accurately indicated the role(s) that these authors had in your study. You can update author roles in the Author Contributions section of the online submission form.

Please also include the following statement within your amended Funding Statement.

“The funder provided support in the form of salaries for authors, but did not have any additional role in the study design, data collection and analysis, decision to publish, or preparation of the manuscript. The specific roles of these authors are articulated in the ‘author contributions’ section.”

If your commercial affiliation did play a role in your study, please state and explain this role within your updated Funding Statement.

b. Please also provide an updated Competing Interests Statement declaring this commercial affiliation along with any other relevant declarations relating to employment, consultancy, patents, products in development, or marketed products, etc.

Within your Competing Interests Statement, please confirm that this commercial affiliation does not alter your adherence to all PLOS ONE policies on sharing data and materials by including the following statement: "This does not alter our adherence to  PLOS ONE policies on sharing data and materials.” (as detailed online in our guide for authors http://journals.plos.org/plosone/s/competing-interests) . If this adherence statement is not accurate and  there are restrictions on sharing of data and/or materials, please state these. Please note that we cannot proceed with consideration of your article until this information has been declared.

Please include both an updated Funding Statement and Competing Interests Statement in your cover letter. We will change the online submission form on your behalf.

4. Thank you for uploading your study's underlying data set. Unfortunately, the repository you have noted in your Data Availability statement does not qualify as an acceptable data repository according to PLOS's standards.

At this time, please upload the minimal data set necessary to replicate your study's findings to a stable, public repository (such as figshare or Dryad) and provide us with the relevant URLs, DOIs, or accession numbers that may be used to access these data. For a list of recommended repositories and additional information on PLOS standards for data deposition, please see https://journals.plos.org/plosone/s/recommended-repositories.

5. We note that Figure 1 in your submission contain map images which may be copyrighted. All PLOS content is published under the Creative Commons Attribution License (CC BY 4.0), which means that the manuscript, images, and Supporting Information files will be freely available online, and any third party is permitted to access, download, copy, distribute, and use these materials in any way, even commercially, with proper attribution. For these reasons, we cannot publish previously copyrighted maps or satellite images created using proprietary data, such as Google software (Google Maps, Street View, and Earth). For more information, see our copyright guidelines: http://journals.plos.org/plosone/s/licenses-and-copyright.

We require you to either present written permission from the copyright holder to publish these figures specifically under the CC BY 4.0 license, or remove the figures from your submission:

a. You may seek permission from the original copyright holder of Figure 1 to publish the content specifically under the CC BY 4.0 license.

We recommend that you contact the original copyright holder with the Content Permission Form (http://journals.plos.org/plosone/s/file?id=7c09/content-permission-form.pdf) and the following text:

“I request permission for the open-access journal PLOS ONE to publish XXX under the Creative Commons Attribution License (CCAL) CC BY 4.0 (http://creativecommons.org/licenses/by/4.0/). Please be aware that this license allows unrestricted use and distribution, even commercially, by third parties. Please reply and provide explicit written permission to publish XXX under a CC BY license and complete the attached form.”

Please upload the completed Content Permission Form or other proof of granted permissions as an "Other" file with your submission.

In the figure caption of the copyrighted figure, please include the following text: “Reprinted from [ref] under a CC BY license, with permission from [name of publisher], original copyright [original copyright year].”

b. If you are unable to obtain permission from the original copyright holder to publish these figures under the CC BY 4.0 license or if the copyright holder’s requirements are incompatible with the CC BY 4.0 license, please either i) remove the figure or ii) supply a replacement figure that complies with the CC BY 4.0 license. Please check copyright information on all replacement figures and update the figure caption with source information. If applicable, please specify in the figure caption text when a figure is similar but not identical to the original image and is therefore for illustrative purposes only.

The following resources for replacing copyrighted map figures may be helpful:

USGS National Map Viewer (public domain): http://viewer.nationalmap.gov/viewer/

The Gateway to Astronaut Photography of Earth (public domain): http://eol.jsc.nasa.gov/sseop/clickmap/

Maps at the CIA (public domain): https://www.cia.gov/library/publications/the-world-factbook/index.html and https://www.cia.gov/library/publications/cia-maps-publications/index.html

NASA Earth Observatory (public domain): http://earthobservatory.nasa.gov/

Landsat: http://landsat.visibleearth.nasa.gov/

USGS EROS (Earth Resources Observatory and Science (EROS) Center) (public domain): http://eros.usgs.gov/#

Natural Earth (public domain): http://www.naturalearthdata.com/

6. If the reviewer comments include a recommendation to cite specific previously published works, please review and evaluate these publications to determine whether they are relevant and should be cited. There is no requirement to cite these works unless the editor has indicated otherwise.

[Note: HTML markup is below. Please do not edit.]

Reviewers' comments:

**Comments to the Author**

1. Is the manuscript technically sound, and do the data support the conclusions?

Reviewer #1: No

**********

2. Has the statistical analysis been performed appropriately and rigorously?

Reviewer #1: No

**********

3. Have the authors made all data underlying the findings in their manuscript fully available?

The PLOS Data policy

Reviewer #1: Yes

**********

4. Is the manuscript presented in an intelligible fashion and written in standard English?

Reviewer #1: Yes

**********

**Reviewer #1:**  The manuscript characterized a multifunctional rhizobacterium, Burkholderia gladioli ST3M-39a, and evaluted its applicability as microbial inoculants in wheat in pot experiment in the greenhouse. The content is fine but to a large extent, its presentation needs a substantial improvement. A major question is its rigorism. The rigor of their research is limited. I list some points for them to consider:

1. The strain is a maize rhizosphere isolate, then why using it in wheat but not in maize for evaluating its growth-promotion?

2. The greenhouse experiment is somewhat the most important part of this work. However, there is no details in section 2.8. No mention of the basic information of soil, e.g., the pH， Pi content, and salt content...

3. In my opinion, if the authors want to claim the multifunction, such as mitigation of salinity, drought, and pH extremes, they should at least measures some representative indicators in wheat.

4. Also, if the conclusion is ST3M-39a matches fertilizer efficacy in wheat via phosphate solubilization, they need to measure the avaiblabe and total Pi contents in soils and plant tissues.

5. Line 199, the positive control is adding 90kg/ha diammonium phosphate, how this can be achieved in pot?

6. Test samples was added 1 mL of phosphate-solubilizing bacterial inoculum, I am wondering how such a small volume was applied? And only once during the whole growing season of wheat?

7. I have no idea how table 1 was composed. How can they get phenotypic features and metabolic properties of these selected isolates based on BLAST?

8. By the way, the colonization and replication of added bacterium in pots should be evaluated to confirm they can really have an effect.

9. In addition, the potential safety issues should be tested or at least discussed before used as a bioinoculant.

10. Line 357, the dry biomass of wheat varied significantly across treatments, ranging from 16.60 to 18.68 gm? What’s the unit “gm” standing for? I can not image the dry weight of a mature wheat seedlings is so light.

**********

what does this mean?). If published, this will include your full peer review and any attached files.

If you choose “no”, your identity will remain anonymous but your review may still be made public.

**Do you want your identity to be public for this peer review?**  For information about this choice, including consent withdrawal, please see our Privacy Policy

Reviewer #1: **Yes:**  Zhongke Sun

**********

[NOTE: If reviewer comments were submitted as an attachment file, they will be attached to this email and accessible via the submission site. Please log into your account, locate the manuscript record, and check for the action link "View Attachments". If this link does not appear, there are no attachment files.]

While revising your submission, please upload your figure files to the Preflight Analysis and Conversion Engine (PACE) digital diagnostic tool, https://pacev2.apexcovantage.com/. PACE helps ensure that figures meet PLOS requirements. To use PACE, you must first register as a user. Registration is free. Then, login and navigate to the UPLOAD tab, where you will find detailed instructions on how to use the tool. If you encounter any issues or have any questions when using PACE, please email PLOS at figures@plos.org. Please note that Supporting Information files do not need this step.

# Author comment: Author response to Decision Letter 1

**DOI:** 10.1371/journal.pone.0332355.r002

1 Jan 2026

Responses to Reviewer Comments

Comment 1: The strain is a maize rhizosphere isolate, then why using it in wheat but not in maize for evaluating its growth-promotion?

Justification: "We thank the reviewer for this pertinent question. The primary rationale for testing this maize rhizosphere isolate on wheat was to assess its broader functional potential as a cross-crop plant growth-promoting rhizobacterium (PGPR). Many PGPR traits, particularly phosphate solubilization, are not strictly host-specific. This approach helps identify robust candidates with wide applicability. We acknowledge that evaluating its efficacy on its native host (maize) is a logical and important next step. We have amended the discussion to clarify this rationale and explicitly state that host-origin testing will be a focus of subsequent research."

Comment 2: The greenhouse experiment is somewhat the most important part of this work. However, there is no details in section 2.8. No mention of the basic information of soil, e.g., the pH， Pi content, and salt content...

Action/Justification: We thank the reviewer for this essential critique. We have now amended Section 2.9 to include detailed reference characteristics of the silty loam soil used, sourced from the same field site. The representative values are: pH 4.76, 2.65% organic matter, 163.35 mg kg⁻¹ P₂O₅, 55.31 mg kg-1 K2O, and 0.12% total nitrogen. We have added a sentence clarifying that these are reference values from the source site, provided to contextualize the experiment, as a full analysis of the specific batch was not conducted. Furthermore, in the Discussion, we now address the relevance of the soil's acidic pH for interpreting the phosphate-solubilizing activity of our isolate.

Comment 3: In my opinion, if the authors want to claim the multifunction, such as mitigation of salinity, drought, and pH extremes, they should at least measures some representative indicators in wheat.

Justification: "We agree with the reviewer and have refined our language to avoid overstatement. The in vitro assays demonstrate the bacterial strain's tolerance to abiotic stresses, which is a desirable trait for a bioinoculant intended for challenging environments. We do not claim, based on the current data, that inoculation mitigates these stresses in plants.

Comment 4: Also, if the conclusion is ST3M-39a matches fertilizer efficacy in wheat via phosphate solubilization, they need to measure the avaiblabe and total Pi contents in soils and plant tissues.

Justification: "This is a valuable and critical point. We acknowledge that measuring available P in the rhizosphere soil and total P in plant tissue at harvest would provide direct mechanistic evidence linking the strain's solubilization activity to plant P uptake. The absence of this data is a limitation of the current study. We have added a paragraph in the Discussion highlighting this limitation and stating that soil/plant P quantification is a prerequisite for advanced field trials."

Comment 5: Line 199, the positive control is adding 90kg/ha diammonium phosphate, how this can be achieved in pot?

Justification: "The field application rate was converted to a pot-scale equivalent based on soil surface area. For a 9-inch (22.86 cm) diameter pot, the surface area is ~0.041 m². The field rate of 90 kg DAP/ha equals 9 g/m². Therefore, the amount per pot was 9 g/m² * 0.041 m² = 0.37 g DAP per pot."

Comment 6: Test samples was added 1 mL of phosphate-solubilizing bacterial inoculum, I am wondering how such a small volume was applied? And only once during the whole growing season of wheat?

Justification: "The 1 mL inoculum was applied as a soil drench near the base of seedlings at the time of thinning. This volume and single application were chosen for the sterile soil system to deliver a precise CFU count while minimizing soil disturbance. We recognize that for field application, a higher volume, carrier-based formulation, or seed treatment would be standard."

Comment 7: I have no idea how table 1 was composed. How can they get phenotypic features and metabolic properties of these selected isolates based on BLAST?

Justification: "We apologize for the lack of clarity. Table 1 presents phenotypic and metabolic properties determined through laboratory assays. We have amended and clarified the caption accordingly."

Comment 8: By the way, the colonization and replication of added bacterium in pots should be evaluated to confirm they can really have an effect.

Justification: "The reviewer raises a fundamental point. We did not monitor the colonization dynamics of ST3M-39a in the wheat rhizosphere in this experiment. Demonstrating successful root colonization is indeed crucial to confirm a direct cause-effect relationship. We have added a statement in the Limitations/Future Work section that 'future studies must employ molecular tools (e.g., qPCR with strain-specific primers or GFP-tagging) to quantify colonization and persistence of the inoculant in the rhizosphere.'"

Comment 9: In addition, the potential safety issues should be tested or at least discussed before used as a bioinoculant.

Justification: " We sincerely thank the reviewer for raising this critical point regarding biosafety, which is essential for the responsible development of any microbial inoculant, particularly for strains within the Burkholderia genus. In direct response to this comment, we have performed two independent safety assessments and have integrated a detailed discussion of the results into the manuscript.

We performed plant pathogenicity assay on bok choy, microscopic examination revealed no necrosis, lesions, or cellular damage in treated leaves. Furthermore, treated plants showed significant increases in leaf length and breadth compared to controls, indicating a growth-promoting rather than pathogenic interaction. Also, the strain exhibited no zone of hemolysis on blood agar, suggesting it lacks this key virulence-associated trait."

Comment 10: Line 357, the dry biomass of wheat varied significantly across treatments, ranging from 16.60 to 18.68 gm? What’s the unit “gm” standing for? I can not image the dry weight of a mature wheat seedlings is so light.

Action/Justification: "1. Unit Correction: 'gm' is a typographical error and has been corrected to the standard unit 'g' (grams) throughout the manuscript.

2. Data Clarification: The reported dry biomass (16.60-18.68 g) is for the total shoot biomass per pot (5 plants) without spikes, not per individual seedling. This has been clarified in the text. We have also double-checked our raw data and measurements to confirm their accuracy."

Comment 11: Problems with statistical analysis

Action/Justification: “We sincerely thank reviewer for pointing out the major errors. All statistical analysis were performed again appropriately and rigorously.”

Attachment Submitted filename: Response to Reviewers.docx

# Peer review: Decision Letter 1

**DOI:** 10.1371/journal.pone.0332355.r003

**Authors:** Vishal Tripathi

21 May 2026

Dear Dr. Pandey,

Thank you for submitting your manuscript to PLOS ONE. After careful consideration, we feel that it has merit but does not fully meet PLOS ONE’s publication criteria as it currently stands. Therefore, we invite you to submit a revised version of the manuscript that addresses the points raised during the review process.

==============================

**ACADEMIC EDITOR:**

- Please address the minor technical comments provided by the reviewer.

Please submit your revised manuscript by Jul 05 2026 11:59PM. If you will need more time than this to complete your revisions, please reply to this message or contact the journal office at plosone@plos.org. When you're ready to submit your revision, log on to https://www.editorialmanager.com/pone/ and select the 'Submissions Needing Revision' folder to locate your manuscript file.

- A letter that responds to each point raised by the academic editor and reviewer(s). You should upload this letter as a separate file labeled 'Response to Reviewers'.
- A marked-up copy of your manuscript that highlights changes made to the original version. You should upload this as a separate file labeled 'Revised Manuscript with Track Changes'.
- An unmarked version of your revised paper without tracked changes. You should upload this as a separate file labeled 'Manuscript'.

If you would like to make changes to your financial disclosure, please include your updated statement in your cover letter. Guidelines for resubmitting your figure files are available below the reviewer comments at the end of this letter.

If applicable, we recommend that you deposit your laboratory protocols in protocols.io to enhance the reproducibility of your results. Protocols.io assigns your protocol its own identifier (DOI) so that it can be cited independently in the future. For instructions see: https://journals.plos.org/plosone/s/submission-guidelines#loc-laboratory-protocols. Additionally, PLOS ONE offers an option for publishing peer-reviewed Lab Protocol articles, which describe protocols hosted on protocols.io. Read more information on sharing protocols at https://plos.org/protocols?utm_medium=editorial-email&utm_source=authorletters&utm_campaign=protocols.

As the corresponding author, your ORCID iD is verified in the submission system and will appear in the published article. PLOS supports the use of ORCID, and we encourage all coauthors to register for an ORCID iD and use it as well. Please encourage your coauthors to verify their ORCID iD within the submission system before final acceptance, as unverified ORCID iDs will not appear in the published article. *Only*  the individual author can complete the verification step; PLOS staff *cannot*  verify ORCID iDs on behalf of authors.

We look forward to receiving your revised manuscript.

Kind regards,

Vishal Tripathi, Ph.D.

Academic Editor

PLOS One

Journal Requirements:

1. If the reviewer comments include a recommendation to cite specific previously published works, please review and evaluate these publications to determine whether they are relevant and should be cited. There is no requirement to cite these works unless the editor has indicated otherwise.

2. Please review your reference list to ensure that it is complete and correct. If you have cited papers that have been retracted, please include the rationale for doing so in the manuscript text, or remove these references and replace them with relevant current references. Any changes to the reference list should be mentioned in the rebuttal letter that accompanies your revised manuscript. If you need to cite a retracted article, indicate the article’s retracted status in the References list and also include a citation and full reference for the retraction notice.

[Note: HTML markup is below. Please do not edit.]

Reviewers' comments:

Reviewer's Responses to Questions

**Comments to the Author**

Reviewer #1: All comments have been addressed

Reviewer #2: All comments have been addressed

**********

2. Is the manuscript technically sound, and do the data support the conclusions?

Reviewer #1: Yes

Reviewer #2: Yes

**********

3. Has the statistical analysis been performed appropriately and rigorously?

Reviewer #1: Yes

Reviewer #2: Yes

**********

4. Have the authors made all data underlying the findings in their manuscript fully available?

The PLOS Data policy

Reviewer #1: Yes

Reviewer #2: Yes

**********

5. Is the manuscript presented in an intelligible fashion and written in standard English?

Reviewer #1: Yes

Reviewer #2: Yes

**********

Reviewer #1: The quality of the revised manuscript is greatly improved. I have a few minor suggestions for the authors.

1. I personally think figure 1 can be moved into supplementary information.

2. Line 225, what’s the cultivar of Triticum aestivum?

3. Line 227, “each replicated 15 times”, what does this mean? How many technical repeats and how many biological replicates?

4. Table 1 can also be moved into supplementary information.

5. Figure 4 should be inserted under line 340 other than line 312.

6. Similar suggestion for the location of figure 5.

7. As the authors have already described the statistics of data in section 2.11, It seems quite distracting to show all F and P values in the results.

8. In section 3.6, is it necessary to show (t (11) = xx, p = xx, η² = xx)?

9. The last paragraph can be deleted, as they are more like expectation by the authors other than conclusion.

Reviewer #2: The manuscript titled "Defying Salinity, Drought, and pH Extremes: A Multifunctional Rhizobacterium, Burkholderia gladioli ST3M-39a, Matches Fertilizer Efficacy in Wheat via Phosphate Solubilization," addresses an important and timely issue and has merit for publication. The paper is accepted subject to minor revisions aimed at improving overall quality and enhancing reader interest. Enhancing figures quality, consistent formatting, and self-explanatory titles, along with detailed statistical analysis in tables, will improve quality.

**********

what does this mean?). If published, this will include your full peer review and any attached files.

If you choose “no”, your identity will remain anonymous but your review may still be made public.

**Do you want your identity to be public for this peer review?**  For information about this choice, including consent withdrawal, please see our Privacy Policy

Reviewer #1: No

Reviewer #2: **Yes:**  Rajesh Kumar Singh

**********

[NOTE: If reviewer comments were submitted as an attachment file, they will be attached to this email and accessible via the submission site. Please log into your account, locate the manuscript record, and check for the action link "View Attachments". If this link does not appear, there are no attachment files.]

To ensure your figures meet our technical requirements, please review our figure guidelines: https://journals.plos.org/plosone/s/figures

You may also use PLOS’s free figure tool, NAAS, to help you prepare publication quality figures: https://journals.plos.org/plosone/s/figures#loc-tools-for-figure-preparation.

NAAS will assess whether your figures meet our technical requirements by comparing each figure against our figure specifications.

# Author comment: Author response to Decision Letter 2

**DOI:** 10.1371/journal.pone.0332355.r004

30 May 2026

Responses to Reviewer Comments

Reviewer #1:

Comment 1: I personally think figure 1 can be moved into supplementary information.

Action: We have moved figure 1 into supplementary information as Fig S1.

Comment 2: Line 225, what’s the cultivar of Triticum aestivum?

Action: We thank reviewer for pointing out the missed information. We have included the cultivar in manuscript as Triticum aestivum ‘WK 1204’.

Comment 3: Line 227, “each replicated 15 times”, what does this mean? How many technical repeats and how many biological replicates?

Action/Justification: This is valuable and critical observation; we thank reviewer for pointing this. We have had 15 biological replicates for pot experiment. We recorded and reported a single set of data for each 15 sets of pots for each treatment group. We have also included “biological replicates” in manuscript.

Comment 4: Table 1 can also be moved into supplementary information.

Action: We have moved Table 1 into supplementary information as S1 Table.

Comment 5 & 6: Figure 4 should be inserted under line 340 other than line 312. Similar suggestion for the location of figure 5.

Action: We thank reviewer for pointing out this error. We have addressed the issue and also, we have made some arrangements for other misplaced figures too.

Comment 7 & 8: As the authors have already described the statistics of data in section 2.11, it seems quite distracting to show all F and P values in the results. In section 3.6, is it necessary to show (t(11) = xx, p = xx, η2 = xx)?

Action/Justification: This is indeed somehow distracting. We have addressed the issues in the manuscript. Also, we have added a supporting information S2 Table including statistical reports.

Comment 9: The last paragraph can be deleted, as they are more like expectation by the authors other than conclusion.

Action: We have deleted the last paragraph.

Reviewer #2: The manuscript titled “Defying Salinity, Drought, and pH Extremes: A Multifunctional Rhizobacterium, Burkholderia gladioli ST3M-39a, Matches Fertilizer Efficacy in Wheat via Phosphate Solubilization”, addresses an important and timely issue and has merit for publication. The paper is accepted subject to minor revisions aimed at improving overall quality and enhancing reader interest. Enhancing figures quality, consistent formatting, and self-explanatory titles, along with detailed statistical analysis in tables, will improve quality.

Action: We thank reviewer for this kind words and impactful suggestions. We have enhanced the figure quality, improved formatting and have provided self-explanatory titles. We have also added an S2 Table in supporting information that detailed the statistical analysis we have carried out.

Attachment Submitted filename: Response to Reviewers V2.docx

# Peer review: Decision Letter 2

**DOI:** 10.1371/journal.pone.0332355.r005

**Authors:** Abhijeet Kashyap

29 Aug 2026

Dear Dr. Pandey,

Thank you for submitting your manuscript to PLOS One. After careful consideration, we feel that it has merit but does not fully meet PLOS One’s publication criteria as it currently stands. Therefore, we invite you to submit a revised version of the manuscript that addresses the points raised during the review process.

Please submit your revised manuscript by Oct 13 2026 11:59PM. If you will need more time than this to complete your revisions, please reply to this message or contact the journal office at plosone@plos.org. When you're ready to submit your revision, log on to https://www.editorialmanager.com/pone/ and select the 'Submissions Needing Revision' folder to locate your manuscript file.

- A letter that responds to each point raised by the academic editor and reviewer(s). You should upload this letter as a separate file labeled 'Response to Reviewers'.
- A marked-up copy of your manuscript that highlights changes made to the original version. You should upload this as a separate file labeled 'Revised Manuscript with Track Changes'.
- An unmarked version of your revised paper without tracked changes. You should upload this as a separate file labeled 'Manuscript'.

If you would like to make changes to your financial disclosure, please include your updated statement in your cover letter. Guidelines for resubmitting your figure files are available below the reviewer comments at the end of this letter.

If applicable, we recommend that you deposit your laboratory protocols in protocols.io to enhance the reproducibility of your results. Protocols.io assigns your protocol its own identifier (DOI) so that it can be cited independently in the future. For instructions see: https://journals.plos.org/plosone/s/submission-guidelines#loc-laboratory-protocols. Additionally, PLOS One offers an option for publishing peer-reviewed Lab Protocol articles, which describe protocols hosted on protocols.io. Read more information on sharing protocols at https://plos.org/protocols?utm_medium=editorial-email&utm_source=authorletters&utm_campaign=protocols.

As the corresponding author, your ORCID iD is verified in the submission system and will appear in the published article. PLOS supports the use of ORCID, and we encourage all coauthors to register for an ORCID iD and use it as well. Please encourage your coauthors to verify their ORCID iD within the submission system before final acceptance, as unverified ORCID iDs will not appear in the published article. *Only*  the individual author can complete the verification step; PLOS staff *cannot*  verify ORCID iDs on behalf of authors.

We look forward to receiving your revised manuscript.

Kind regards,

Abhijeet Shankar Kashyap

Academic Editor

PLOS One

Journal Requirements:

1. If the reviewer comments include a recommendation to cite specific previously published works, please review and evaluate these publications to determine whether they are relevant and should be cited. There is no requirement to cite these works unless the editor has indicated otherwise.

[Note: HTML markup is below. Please do not edit.]

Reviewer's Responses to Questions

**Comments to the Author**

Reviewer #2: All comments have been addressed

**********

2. Is the manuscript technically sound, and do the data support the conclusions?

Reviewer #2: Yes

**********

3. Has the statistical analysis been performed appropriately and rigorously?

Reviewer #2: Yes

**********

4. Have the authors made all data underlying the findings in their manuscript fully available?

The PLOS Data policy

Reviewer #2: Yes

**********

5. Is the manuscript presented in an intelligible fashion and written in standard English?

Reviewer #2: Yes

**********

Reviewer #2: The authors have carefully addressed all of the reviewers' comments in a thorough and satisfactory manner. The revisions have significantly improved the clarity, scientific rigor, and overall quality of the manuscript. The responses provided are well-reasoned, comprehensive, and adequately justify the changes made throughout the revised version.

But some minor comments have been mentioned below.

2.1. Protocol availability:

Line No. 91The protocols used in this study are available in protocols.io with DOI: dx.doi.org/10.17504/protocols.io.yxmvm17j6v3p/v1.

Please mention the name of the study to see the protocol availability

2.2 Materials and equipment:

Replaced with Chemical and Equipment

Figure 3 add the absorbance OD600 in x-axis.

Figure 2 & 4 appears blurry. Please use a clearer, high-resolution version.

**********

what does this mean?). If published, this will include your full peer review and any attached files.

If you choose “no”, your identity will remain anonymous but your review may still be made public.

**Do you want your identity to be public for this peer review?**  For information about this choice, including consent withdrawal, please see our Privacy Policy

Reviewer #2: **Yes:**  Rajesh Kumar Singh

**********

[NOTE: If reviewer comments were submitted as an attachment file, they will be attached to this email and accessible via the submission site. Please log into your account, locate the manuscript record, and check for the action link "View Attachments". If this link does not appear, there are no attachment files.]

To ensure your figures meet our technical requirements, please review our figure guidelines: https://journals.plos.org/plosone/s/figures

You may also use PLOS’s free figure tool, NAAS, to help you prepare publication quality figures: https://journals.plos.org/plosone/s/figures#loc-tools-for-figure-preparation.

NAAS will assess whether your figures meet our technical requirements by comparing each figure against our figure specifications.

# Author comment: Author response to Decision Letter 3

**DOI:** 10.1371/journal.pone.0332355.r006

19 Sep 2026

To,

The Editor

PLOS ONE

Subject: Revised Manuscript Submission - PONE-D-25-47295R2

Title: Defying Salinity, Drought, and pH Extremes: A Multifunctional Rhizobacterium, Burkholderia gladioli ST3M-39a, Matches Fertilizer Efficacy in Wheat via Phosphate Solubilization

Dear Editor,

Please find enclosed our revised manuscript (Manuscript ID: PONE-D-25-47295R2), which we are resubmitting for consideration for publication in PLOS ONE.

We are grateful to the editor and reviewer for the thoughtful and constructive feedback, which has greatly strengthened our manuscript. We have carefully addressed all comments and concerns in full. Key revisions include:

1. 2.1 protocol availability: Line No. 91 The protocols used in this study are available in protocols.io with DOI: dx.doi.org/10.17504/protocols.io.yxmvm17j6v3p/v1, please mention the name of the study to see the protocol availability: We have mentioned the name of the study in protocol availability section as suggested.

2. 2.2 Materials and equipment: replaced with Chemical and Equipment: As suggested, the heading has been changed to “Chemical and Equipment”.

3. Figure 3 add the absorbance OD600 in x-axis: We have corrected the error and have added OD600 to the x-axis.

4. Figure 2 & 4 appears blurry, please use a clearer, high-resolution version: We have uploaded the clearer, high-resolution versions of these figures.

All changes in the manuscript are highlighted for your convenience. We hope the revised manuscript is now suitable for publication in PLOS ONE. Thank you for your efforts in improving this manuscript.

With Best Regards,

Gyanu Raj Pandey

Corresponding Author

Shubham Biotech Nepal Private Limited

Email: gyanupandey9@gmail.com

Phone number: +9779860470938

Attachment Submitted filename: Response to Reviewers.pdf

# Editor report: Decision Letter 3

**DOI:** 10.1371/journal.pone.0332355.r007

**Authors:** Abhijeet Kashyap

24 Sep 2026

Defying Salinity, Drought, and pH Extremes: A Multifunctional Rhizobacterium, Burkholderia gladioli ST3M-39a, Matches Fertilizer Efficacy in Wheat via Phosphate Solubilization

PONE-D-25-47295R3

Dear Dr. Pandey,

We’re pleased to inform you that your manuscript has been judged scientifically suitable for publication and will be formally accepted for publication once it meets all outstanding technical requirements.

Within one week, you’ll receive an e-mail detailing the required amendments. When these have been addressed, you’ll receive a formal acceptance letter and your manuscript will be scheduled for publication.

An invoice will be generated when your article is formally accepted. Please note, if your institution has a publishing partnership with PLOS and your article meets the relevant criteria, all or part of your publication costs will be covered. Please make sure your user information is up-to-date by logging into Editorial Manager at Editorial Manager® and clicking the ‘Update My Information' link at the top of the page. For questions related to billing, please contact billing support.

If your institution or institutions have a press office, please notify them about your upcoming paper to help maximize its impact. If they’ll be preparing press materials, please inform our press team as soon as possible -- no later than 48 hours after receiving the formal acceptance. Your manuscript will remain under strict press embargo until 2 pm Eastern Time on the date of publication. For more information, please contact onepress@plos.org.

Kind regards,

Abhijeet Shankar Kashyap

Academic Editor

PLOS One

Additional Editor Comments (optional):

Reviewers' comments:

# Editor report: Acceptance letter

**DOI:** 10.1371/journal.pone.0332355.r008

**Authors:** Abhijeet Kashyap

PONE-D-25-47295R3

PLOS One

Dear Dr. Pandey,

I'm pleased to inform you that your manuscript has been deemed suitable for publication in PLOS One. Congratulations! Your manuscript is now being handed over to our production team.

At this stage, our production department will prepare your paper for publication. This includes ensuring the following:

* All references, tables, and figures are properly cited

* All relevant supporting information is included in the manuscript submission,

* There are no issues that prevent the paper from being properly typeset

You will receive further instructions from the production team, including instructions on how to review your proof when it is ready. Please keep in mind that we are working through a large volume of accepted articles, so please give us a few days to review your paper and let you know the next and final steps.

Lastly, if your institution or institutions have a press office, please let them know about your upcoming paper now to help maximize its impact. If they'll be preparing press materials, please inform our press team within the next 48 hours. Your manuscript will remain under strict press embargo until 2 pm Eastern Time on the date of publication. For more information, please contact onepress@plos.org.

You will receive an invoice from PLOS for your publication fee after your manuscript has reached the completed accept phase. If you receive an email requesting payment before acceptance or for any other service, this may be a phishing scheme. Learn how to identify phishing emails and protect your accounts at https://explore.plos.org/phishing.

If we can help with anything else, please email us at customercare@plos.org.

Thank you for submitting your work to PLOS One and supporting open access.

Kind regards,

PLOS One Editorial Office Staff

on behalf of

Dr. Abhijeet Shankar Kashyap

Academic Editor

PLOS One
